# Global land-use change rivals climate change in driving projected diversity shifts of terrestrial vertebrates

**DOI:** 10.1016/j.isci.2026.117148

**Published:** 2026-08-10

**Authors:** Xiaojuan Liu, Bingqi Xie, Wei Tu, Yang Wu, Minyi Gao, Nan Xu, Xia Li

**Affiliations:** 1 School of Architecture and Urban Planning, Shenzhen University, 3688 Nanhai Avenue, Nanshan District, Shenzhen, Guangdong 518060, P.R. China; 2 Key Laboratory of Geographic Information Science (Ministry of Education), School of Geographic Sciences, East China Normal University, Shanghai 200241, P.R. China; 3 Key Laboratory for Geo-Environmental Monitoring of Great Bay Area, Guangdong Key Laboratory for Urban Informatics, Shenzhen University, Shenzhen 518060, P.R. China; 4 State Key Laboratory of Subtropical Building and Urban Science, Shenzhen 518060, P.R. China; 5 Shenzhen Key Laboratory of Spatial Information Smart Sensing and Services, and Department of Urban Informatics, School of Architecture and Urban Planning, Shenzhen University, 3688 Nanhai Avenue, Nanshan District, Shenzhen, Guangdong 518060, P.R. China; 6 Guangdong Key Laboratory of Animal Conservation and Resource Utilization, Guangdong Public Laboratory of Wild Animal Conservation and Utilization, Institute of Zoology, Guangdong Academy of Sciences, Guangzhou, Guangdong 510260, P.R. China

**Keywords:** biodiversity projection, land-use change, climate change, terrestrial vertebrates

## Abstract

Global biodiversity is declining at an unprecedented rate under the combined pressures of climate and land-use change, yet their relative contributions and underlying mechanisms remain poorly understood. Here, we developed a filtering-energy-stress framework to characterize multidimensional land-use change and integrate it, together with climate change, into species distribution models for projecting future biodiversity dynamics under shared socio-economic pathways (SSPs). We found that 68.14% of terrestrial land was projected to experience richness declines by 2100, with land-use change contributing 47.94% of total changes, comparable to climate change (52.06%). The Shapley additive explanations (SHAP) analysis revealed that available energy was the most influential land-use dimension across vertebrate taxa, outperforming habitat suitability and land-use intensity. These findings underscore the importance of considering multiple ecological dimensions of land-use change beyond land-cover categories and provide a mechanistic framework for disentangling climate and land-use impacts to inform integrated conservation strategies.

## Highlights

• A filtering-energy-stress (FES) framework is built to capture land-use change
• By 2100, 68.14% of global land is projected to have vertebrate richness declines
• Land-use change explains 47.94% of projected biodiversity change globally
• Available energy dominates land-use effects on future biodiversity patterns

## Graphical abstract

**Figure:**
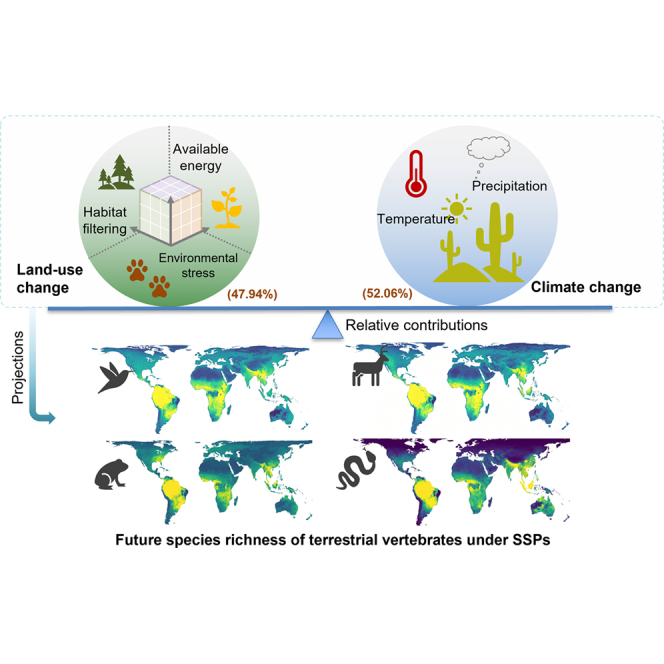

## Teaser

Environmental science; Global change; Terrestrial science; Biogeoscience

## Introduction

Global biodiversity, often measured through species richness, plays a crucial role in maintaining ecosystem functions and human welfare.^1^ Yet biodiversity on Earth has been declining steadily since the Holocene, posing a significant threat to global sustainable development goals.^2^ The World Economic Forum (WEF) has identified “biodiversity loss” as a top-tier global economic risk in the coming decades,^3^ further underscoring the urgency of effective conservation efforts to turn this declining trend. However, actionable conservation strategies require a holistic understanding of how biodiversity responds to driving factors of its decline. Terrestrial vertebrates serve as essential indicators of global biodiversity, supporting food security and pharmaceutical development through regulating critical ecological processes, including decomposition, carbon cycling, and pollination.^4^^,^^5^ Their shifts in range and declines in diversity not only reflect broad systemic collapse within ecosystems, but also capture the combined effects of potential drivers (e.g., land-use and climate change).^6^^,^^7^ Therefore, establishing a comprehensive understanding of terrestrial vertebrate species responses to their drivers is essential for identifying priority areas for global biodiversity conservation and informing sustainable spatial planning.

Diversity loss of terrestrial vertebrates is driven by multifaceted factors, encompassing both natural processes and anthropogenic activities.^8^ Climate change has been widely recognized as a primary driver of future species ranges, as rising temperatures and intensifying precipitation extremes profoundly narrow the climatic niches of vertebrate species.^9^^,^^10^^,^^11^^,^^12^ Nevertheless, climate change rarely acts in isolation. Land-use change constitutes a parallel driver that frequently interacts with climate change to compound terrestrial vertebrate diversity loss.^9^^,^^13^^,^^14^^,^^15^ Such interactions can probably cause habitat degradation, fragmentation, and permanent loss, especially in climate-sensitive regions.^16^ The interacting effects are spatially heterogeneous since different land-use types alter habitats in different ways and to different degrees. For instance, urban expansion in global drylands, where ecosystems are highly vulnerable to climate change, has caused significant habitat degradation and affected approximately 60% of threatened species that depend on natural dryland habitats over recent decades.^17^ Similarly, agricultural expansion and urban sprawl in wetland areas intensify climate-driven losses of soil organic carbon, undermining both ecosystem functioning and species diversity.^18^ These heterogeneous consequences highlight the need to understand the ecological mechanisms through which different land-use changes influence biodiversity responses.

The mechanisms underlying the heterogeneous effects of land-use change on vertebrate diversity are closely associated with three complementary ecological hypotheses: (1) the habitat filtering hypothesis, which posits that land-use types function as environmental filters.^19^^,^^20^ These filters selectively permit the persistence of species whose niche requirements are compatible with the prevailing habitat conditions. As a result, species-specific habitat suitability reflects the extent to which a land-use type provides the environmental conditions necessary for occurrence and persistence; (2) the available energy hypothesis, which contends that landscapes with greater resource availability can support higher species diversity.^21^^,^^22^ For instance, among landscapes with similar habitat types, areas with higher vegetation productivity provide more food resources and available energy to sustain a greater species richness than areas with lower productivity^23^; (3) the environmental stress hypothesis, which emphasizes that biodiversity is constrained by the intensity of different land-use types.^24^^,^^25^ The land-use intensity can directly reduce species persistence through habitat degradation, fragmentation, and other human pressures even where the land-use type remains unchanged. Elevated levels of land-use intensity can alter habitat conditions, reduce resource acquisition, and increase extinction risk.^26^^,^^27^ Although many studies have provided empirical evidence to support each of these hypotheses,^19^^,^^27^^,^^28^ they have largely been investigated independently at the global scale. The integrated effects of land-use change on vertebrate diversity through habitat suitability, energy availability, and land-use intensity remain unclear. This challenge partly arises from the potential interactions among these mechanisms, as energy availability and environmental stress may influence habitat suitability. Nevertheless, each mechanism can also independently affect species persistence beyond habitat filtering alone. These hypotheses can collectively represent complementary and parallel mechanisms that provide a basis for understanding multidimensional land-use impacts on biodiversity.

This knowledge gap is particularly evident in future biodiversity projections, as the multidimensional nature of land-use change is rarely incorporated into projection models.^29^ Numerous studies have reported that the long-term impacts of land-use change on future biodiversity loss may rival or even exceed those of climate change, yet these projections typically treat land-use types as simple categorical variables.^6^^,^^30^^,^^31^ Moreover, future biodiversity change varies considerably among regions and taxonomic groups, likely reflecting differences in habitat filtering, energy availability, and land-use intensity across land-use types. Yet whether and how these mechanisms jointly shape the future distribution of terrestrial vertebrates at the global scale remains poorly understood.

To fill this gap, we developed a three-dimensional land-use framework that explicitly characterizes habitat filtering, available energy, and land-use intensity across landscapes. By integrating this framework into species distribution models (SDMs), we projected species richness of terrestrial vertebrates in response to global land-use and climate change under the SSP scenarios. Specifically, we aimed to answer two questions: (1) how will global terrestrial vertebrate species richness change under future climate and land-use change scenarios? and (2) to what extent does incorporating the multidimensional nature of land-use change alter projections of future terrestrial vertebrate species richness? The insights provided by this study can facilitate the identification of global priority areas for targeted conservation and contribute to the achievement of the Kunming-Montreal Global Biodiversity Framework.

## Results

### Global land filtering-energy-stress patterns

Forests constitute the primary habitat for the majority of terrestrial vertebrate species despite their diverse habitat preferences. Based on species range maps, approximately 80% of amphibian ranges overlap with forest ecosystems, followed by birds (63.50%). Bird range areas also include substantial proportions of cropland (16.92%), grassland (7.80%), and aquatic habitats (6.15%). In addition to forest ecosystems, reptile range maps include relatively large proportions of barren land (8.85%) and grassland (7.79%), whereas mammal range maps encompass substantial areas of cropland (17.43%) and grassland (7.80%). Consistent with various habitat compositions, land-use suitability varies significantly across taxonomic groups, with birds exhibiting the highest suitability and amphibians the lowest. This taxonomic disparity extends to the projected changes in land-use suitability, where birds experience the most substantial decline in average land-use suitability index (LSI) while amphibians face the most moderate reduction. The contrasting responses likely stem from disparities in habitat composition and thereby varying levels of exposure to land-use transitions. Taxa with a greater proportion of forest habitats within their range maps generally exhibit smaller reductions in LSI, suggesting that forest-dominated ranges may be less vulnerable to future land-use change. The average LSI is projected to decline under all SSP scenarios by 2100. For instance, the average LSI of bird species is expected to decline by 29.70% and 32.20% in SSP1 and SSP3, respectively. Comparable scenario-dependent responses were observed in amphibians, mammals, and reptiles, with average LSI consistently highest under SSP1 and lowest under SSP3.

**Figure 1:**
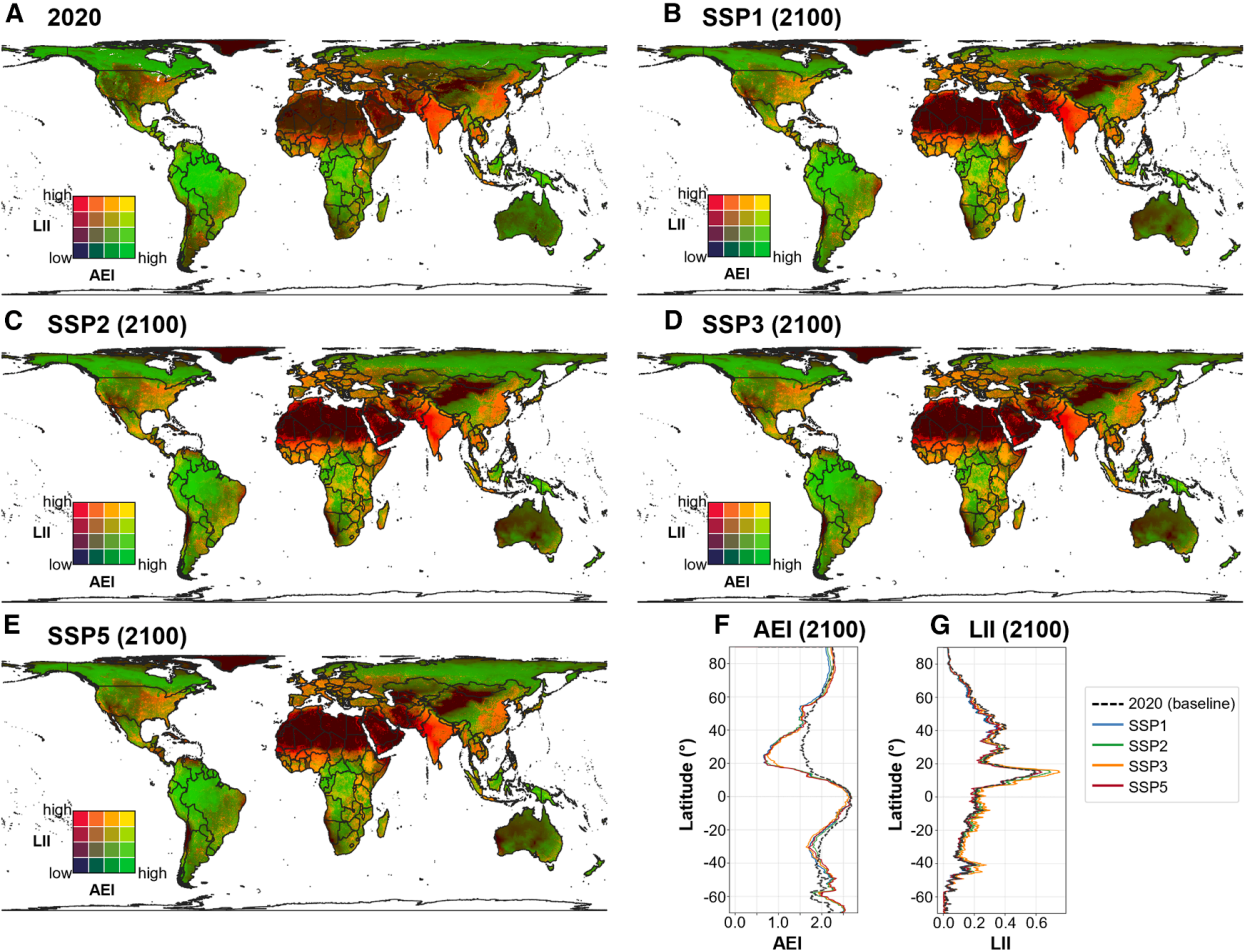
Global maps and latitudinal gradients of the available energy index and land-use intensity index (A–E) Spatial patterns of AEI and LII in 2020 and four SSP scenarios of 2100. (F) Latitudinal distribution of AEI in 2100. (G) Latitudinal distribution of LII in 2100.

The spatial distribution of land energy availability and land-use intensity in 2020 exhibits marked geographic heterogeneity, with both indicators concentrated in low-latitude regions. As shown in Figure 1A, the latitude-weighted centroid of the available energy index (AEI) is positioned closer to the equator (∼5.63°N) than that of the land-use intensity index (LII) (∼14.81°N). Notably, tropical regions record high AEI and LII values, which indicate a strong spatial overlap between areas of high ecological potential and intense land-use pressure. Future AEI is projected to remain below the 2020 baseline in all four SSPs, with the most significant reduction of 10.67% occurring in SSP3 (Figure 1F). Although these scenarios show declining trends, the AEI exhibits relatively limited inter-scenario divergence. The latitude-weighted centroid of the AEI shifts southward in every scenario, indicating a redistribution of ecosystem energy toward equatorial regions. In contrast, LII shows substantially stronger sensitivity to socioeconomic pathways and pronounced scenario-dependent divergence. SSP1 and SSP5 maintain relatively lower levels by 2100, whereas SSP3 is characterized by a persistent increase, reaching 12.0% above the 2020 baseline and exceeding SSP1 and SSP5 by 20.8% and 19.0%, respectively. This increase is particularly evident in tropical regions, where mean LII rises by 17.42%. The southward shift of the LII centroid across all scenarios similarly indicates a redistribution of anthropogenic pressure toward lower latitudes.

### Projected vertebrate diversity under different SSP scenarios

**Figure 2:**
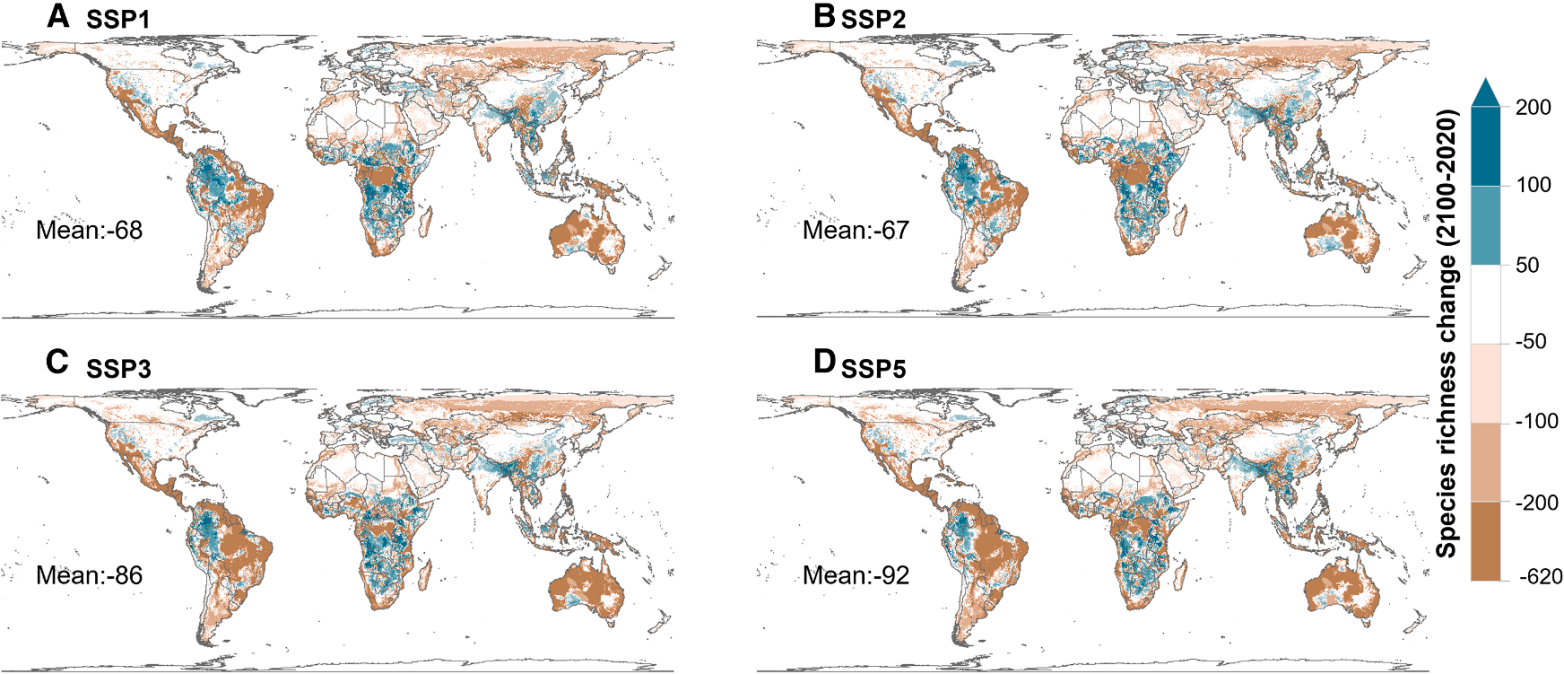
Global maps of species richness changes between 2020 and 2100 Projected species richness changes of terrestrial vertebrates at the grid-cell level between 2020 and 2100 across (A) SSP1, (B) SSP2, (C) SSP3, and (D) SSP5.

By 2100, climate and land-use change are projected to cause widespread terrestrial vertebrate richness loss worldwide (Table 1). On average across SSP scenarios, 68.14% of global land is expected to experience species richness declines, whereas richness gains occur in 31.32% of land areas and only 0.55% remains relatively stable. Spatially, richness losses are concentrated across tropical and subtropical biodiversity hotspots, particularly in the Amazon Basin, the Congo Basin, Southeast Asia, and northern Australia, whereas richness gains are primarily projected in high-latitude regions, especially across northern North America and Eurasia (Figure 2). The most severe declines occur within tropical regions, where exceptionally high baseline species richness amplifies the magnitude of biodiversity loss. The Amazon Basin emerges as the most vulnerable region, accounting for most of the strongest loss hotspots globally, while substantial declines are also projected for the Congo Basin and Southeast Asian tropical forests. In contrast, richness gains in northern high latitudes likely reflect poleward range expansions into newly suitable habitats. These projected trends align with global assessments,^7^^,^^32^ which identify tropical and Mediterranean biomes as exhibiting disproportionate sensitivity to the combined pressures of land-use and climate change. Furthermore, the identified vulnerability of tropical regions is consistent with the findings of Chaudhary and Mooers,^33^ whose land-use change scenarios similarly pinpoint these areas as primary hotspots of vertebrate biodiversity loss. Although the overall spatial patterns are broadly consistent among SSPs, the magnitude of biodiversity change differs substantially across scenarios. SSP5 produces the most severe biodiversity decline, with 71.93% of global land experiencing richness loss and a net richness loss of 92 species per grid cell, followed by SSP3 (69.89%, 86 species). In contrast, SSP2 exhibits the least extensive decline, affecting 64.97% of global land and resulting in a net richness loss of 67 species per grid cell, while SSP1 shows a similar but slightly stronger decline (65.75%, 68 species). The greater losses under SSP3 and SSP5 are particularly evident in tropical regions, where extensive reductions in species richness occur across the Amazon Basin, central Africa, and northern Australia. These results indicate that future biodiversity outcomes are highly sensitive to socioeconomic pathways, with scenarios characterized by greater environmental pressure leading to more widespread and severe richness loss.

**Table 1:** Percentage (%) of terrestrial land with species richness change by 2100 across different SSPs

Taxonomic groups		SSP1	SSP2	SSP3	SSP5	Average
Total	loss	65.75	64.97	69.89	71.93	68.14
	gain	33.67	34.48	29.58	27.55	31.32
	stable	0.59	0.56	0.54	0.52	0.55
Amphibians	loss	59.79	59.47	64.67	67.34	62.82
	gain	25.73	25.82	21.65	19.78	23.25
	stable	14.48	14.71	13.68	12.88	13.94
Mammals	loss	59.05	58.56	64.10	66.10	61.95
	gain	38.19	38.76	33.48	31.44	35.47
	stable	2.76	2.69	2.42	2.46	2.58
Birds	loss	66.97	65.98	70.51	72.20	68.92
	gain	32.19	33.16	28.72	27.05	30.28
	stable	0.84	0.85	0.78	0.75	0.81
Reptiles	loss	61.54	61.10	66.51	68.85	64.50
	gain	30.33	30.65	25.78	23.74	27.63
	stable	8.14	8.25	7.71	7.41	7.88

The percentage of total richness loss is calculated based on changes in the total number of amphibian, mammal, bird, reptile species occurring within each grid cell. A grid cell is considered to experience richness loss when the combined species richness of all terrestrial vertebrates declined relative to the baseline period.

**Figure 3:**
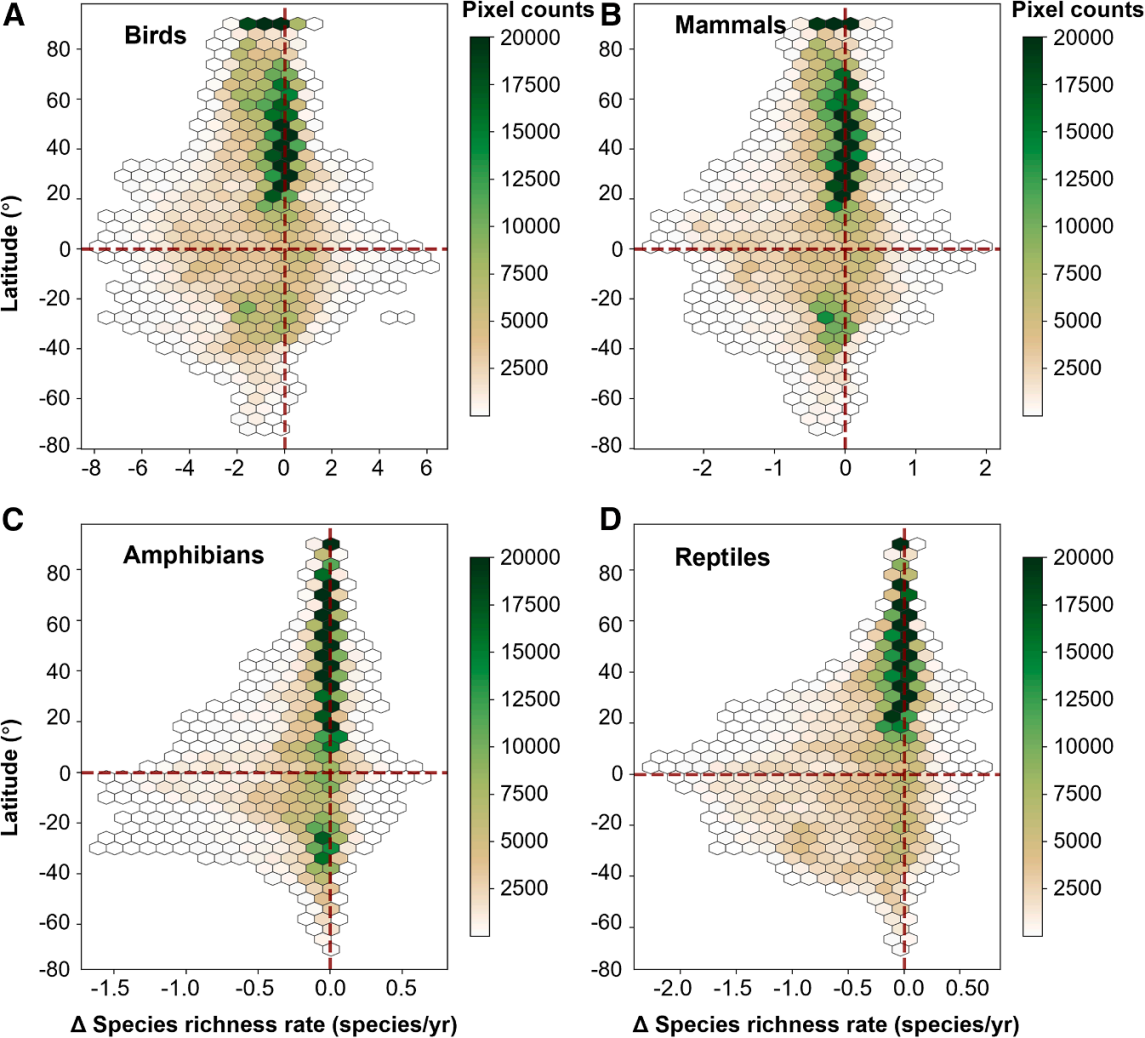
Annual rate of species richness change across geographic latitudes from 2020 to 2100 under SSP5 (A) birds, (B) mammals, (C) amphibians, and (D) reptiles. The calculation is based on the grid-cell level. The color scale shows the density of grid cells for a given annual rate of change and latitude. Red dashed lines denote the zero-change threshold (vertical) and the Equator (horizontal).

Richness changes exhibit a pronounced latitudinal gradient, with losses concentrated at low latitudes (30°S–30°N) and modest gains emerging only at high northern latitudes. For instance, the tropical bands experience declines of up to 158 species per grid cell under SSP5 by 2100, with approximately 54% of cells suffering severe decline (100 species loss), while only high northern latitudes (60–90°N) show a notable proportion of gain (16.70% of grid cells, 49.70 species gain). These latitudinal patterns are consistent across all SSP scenarios, though the magnitude of loss intensifies markedly under higher-emission pathways. More specifically, all four taxonomic groups exhibit losses concentrated at low latitudes, but with notable differences in extent and magnitude (Figure 3; Figures S2–S5). Birds show the most widespread impact, with 72.20% of global land experiencing loss at a mean decline of 62 species per cell and the largest richness change rate among all groups (up to 8 species loss per year) by 2100. Amphibian losses are concentrated in low-latitude areas such as the Amazon Basin and the southern Congo Basin, and accelerate most rapidly over time, expanding from 57.76% of land area in 2040 to 67.28% in 2100, reflecting their high climate sensitivity and limited dispersal capacity. Mammal losses are more diffusely distributed across tropical and subtropical regions, yet mammals show the highest proportion of richness gain areas at 31.4%, suggesting greater range-shift potential at high latitudes. Reptile losses concentrate in Australia and the tropical Americas. These results show a whole range shift from low latitudes to high latitudes as a result of climate and land-use change, coincident with those of previous studies.^34^^,^^35^^,^^36^ Compared with amphibians and reptiles, mammals exhibit more negative species-richness change rates across many latitudinal bands (Figure 3). Furthermore, our projections find that the loss of species richness is concentrated at low altitudes (<1,500 m). Taking SSP5 for example, the largest species richness loss below 1500 m reached five species per year, with birds experiencing the largest loss, followed by mammals (Figure S6).

**Figure 4:**
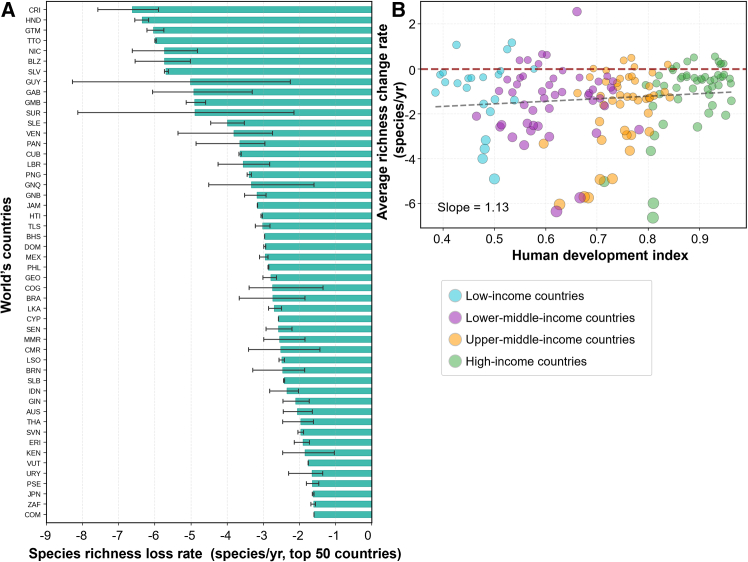
Species richness change in terrestrial vertebrates at the country level from 2020 to 2100 (A) The species richness loss rate for the top 50 countries in the world. Black solid lines indicate the 95% confidence interval of country-specific richness loss. Labels in the vertical axis are the country code (ISO3) of the top 50 countries in the world. (B) Average richness change rate in relation to countries’ human development index (HDI). Colors represent the countries in different income levels and the point diameter indicates the rate of species richness change. The black dashed line (slope = 1.13) represents the linear regression fitted across all countries, whereas the red dashed line indicates the zero reference line.

At the country level, species richness losses are projected to be widespread, with 85.80% of countries experiencing net decline on average across SSP scenarios (Figure 4). Upper-middle-income countries face the most severe losses among losing nations (1.94 species loss/year), followed by lower-middle-income countries (1.68 species loss/year), while high-income and low-income countries show comparatively moderate declines (1.32 and 1.35 species loss/year, respectively). The most severely affected countries are concentrated in Central America and West Africa, led by Costa Rica (6.63 species loss/year), Honduras (6.35 species loss/year), and The Gambia (4.90 species loss/year). In contrast, countries with net richness gains are largely located at higher latitudes and in parts of Africa and South Asia, including Sweden (0.23 species gain/year), Rwanda (1.17 species gain/year), and Bangladesh (2.55 species gain/year). Losses further intensify under higher emission pathways, with the country-level mean declining from 1.18 species loss per year under SSP1 to 1.50 species loss per year under SSP5.

### Relative contributions of land-use and climate change to future vertebrate diversity

**Figure 5:**
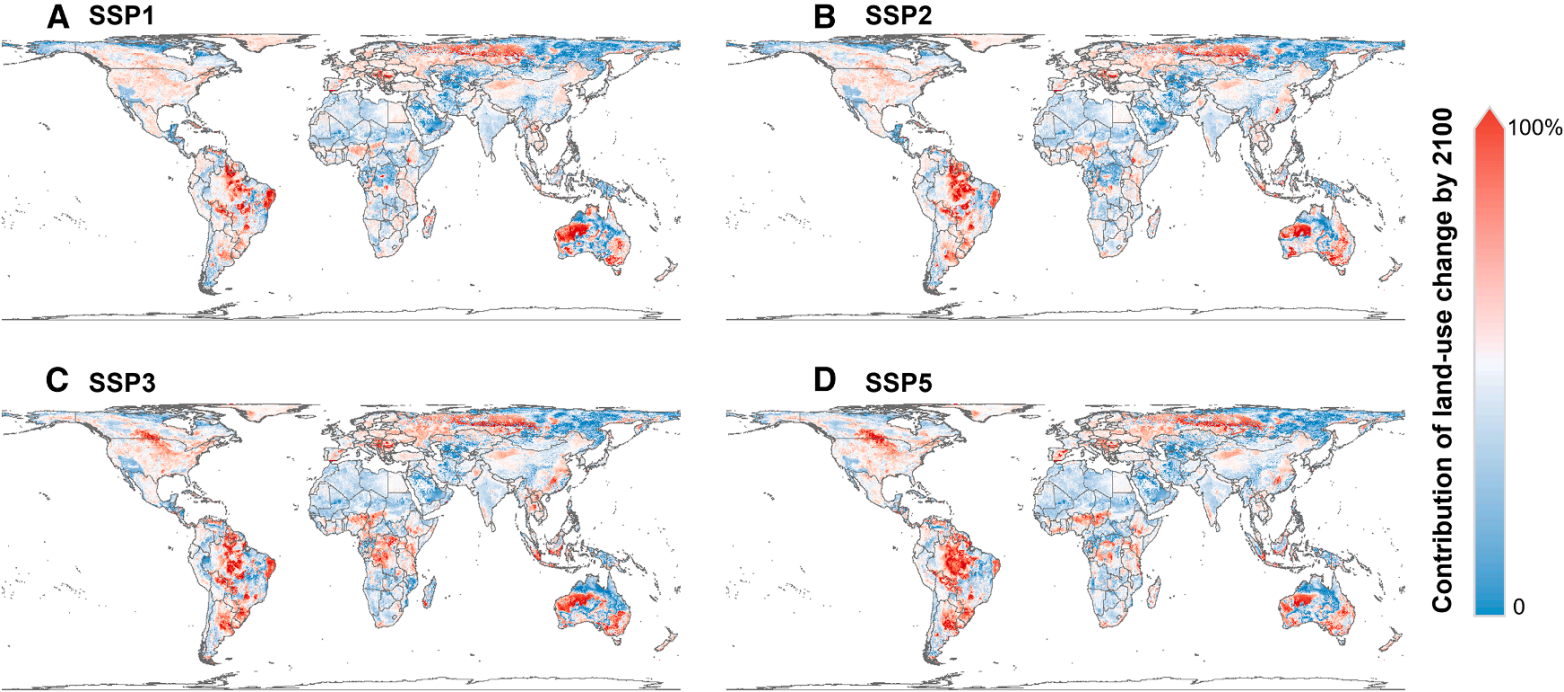
Contribution of future land-use change on global vertebrate species changes (A) SSP1, (B) SSP2, (C) SSP3, and (D) SSP5.

To disentangle the relative contributions of climate change and land-use change to projected richness diversity, we decomposed total richness change into its two drivers across all scenarios and taxa. Overall, climate change is the dominant driver, accounting for a mean contribution of 52.06% across all scenarios and taxa, compared to 47.94% for land-use change. Nevertheless, land-use change plays an increasingly important role over time, with its relative contribution rising from 48.53% in 2040 to 49.34% in 2100 under SSP5. Similar increasing trends are also observed under SSP3 (from 48.56% to 49.15%). This result suggests that land-use change may rival climate change as a driver of biodiversity loss by 2100 across high-pressure SSP pathways. Geographically, land-use change contributes most strongly to projected richness change in tropical regions, particularly in the Amazon Basin, Central Africa, and Southeast Asia (Figure 5). These regions are characterized by extensive land-use transitions, which may contribute to a greater contribution of land-use change relative to climate change. Additional hotspots of high land-use contribution are consistently observed in eastern Australia across all four SSPs. By contrast, climate change contributes more to projected richness change in high-latitude regions and across many arid and semi-arid regions (Figure S7). This pattern is particularly evident in northern North America and northern Eurasia, as well as parts of Central Asia and inland Australia. Among taxonomic groups, birds and reptiles are most sensitive to climate change, accounting for 52.36% and 51.60% of projected richness change, respectively. In contrast, amphibians and mammals show nearly equal contributions from climate and land-use change, with climate change explaining only 50.10% and 50.09% of projected richness change across most of their ranges, respectively.

To further assess the relative influence of multidimensional land-use predictors in shaping projected richness changes, we applied SHAP to calculate the mean absolute contribution of each predictor across species distribution models (Table 2). Across all terrestrial vertebrates, the AEI emerged as the dominant land-use predictor (mean |SHAP|: 0.74), outperforming both the LSI (0.60) and the LII (0.47). Among all predictive variables, AEI ranked second only to the first principal component of bioclimatic variables (PC1: 0.91), underscoring its exceptional importance relative to other land-use dimensions. Collectively, the three land-use indices accounted for a combined contribution comparable to that of all four climate principal components, highlighting the substantial and aggregate role of land-use forcing in driving projected richness changes.

**Table 2:** Mean absolute SHAP values of biodiversity drivers for projected species richness changes of terrestrial vertebrates under climate and land-use change

Variables	Total	Amphibians	Birds	Mammals	Reptiles
LSI	0.60	0.23	0.93	0.63	0.36
AEI	0.74	0.37	1.16	0.75	0.43
LII	0.47	0.19	0.75	0.49	0.29
PC1	0.91	0.45	1.39	0.95	0.61
PC2	0.51	0.24	0.75	0.53	0.35
PC3	0.44	0.23	0.66	0.46	0.30
PC4	0.50	0.22	0.77	0.52	0.31
Elevation	0.41	0.18	0.69	0.48	0.24

LSI, AEI, and LII are land-use suitability, available energy, and land-use intensity indices, respectively. PC1 and PC4 predominantly represent the temperature gradient and extremes, respectively. PC2 and PC3 indicate the precipitation gradient and seasonality, respectively. Elevation: topographic elevation. Values represent the mean absolute SHAP contribution of each predictor to the species distribution models.

The SHAP-derived feature importance exhibited pronounced taxonomic heterogeneity across vertebrate groups. Among the three land-use dimensions, AEI consistently emerged as the most influential variables, with the highest importance observed in birds, followed by mammals, reptiles, and amphibians (Table 2). Similar taxonomic patterns were observed for habitat suitability (LSI) and LII. Despite variation in magnitude, the relative ranking of land-use dimensions was conserved across taxa (AEI > LSI > LII), indicating a consistent hierarchy of land-use effects on projected biodiversity change. Specifically, birds generally exhibited higher SHAP values than the other vertebrate groups for all three land-use indicators, whereas amphibians and reptiles showed comparatively lower values. These results indicate that available energy, habitat suitability, and land-use intensity contribute to biodiversity projections in a consistent hierarchical manner across taxonomic groups, with available energy representing the dominant land-use index.

## Discussion

Climate and land-use change are widely recognized as the two dominant drivers of contemporary biodiversity loss, yet the ecological mechanisms through which land-use change influences biodiversity remain insufficiently understood. To address this gap, we developed a filtering-energy-stress (FES) framework that translates land-use change into three mechanistic ecological dimensions, namely, habitat filtering, available energy, and environmental stress, and integrated them with species distribution models to project future biodiversity change. Our results uncover that climate change has been demonstrated to be the dominant driver of projected changes in terrestrial vertebrate diversity, accounting for 52.06% of the total projected biodiversity change. The SHAP-based importance analysis further reveals that the first principal component of the bioclimatic variables, which predominantly represents the temperature gradient, exerts the greatest influence on the richness changes of terrestrial vertebrates. This is consistent with previous studies that measured the effects of climate change from different perspectives.^37^^,^^38^ Climate change contributes disproportionately to projected biodiversity change in high-latitude regions and parts of arid and semi-arid regions. This pattern is consistent with recent evidence showing that high-latitude regions have experienced more rapid warming than tropical areas, leading to stronger climate-driven shifts in species distributions.^39^ In arid and semi-arid regions, low soil moisture amplifies heat stress through limited evaporative cooling and therefore intensifies the sensitivity of vertebrate species to climatic variation.^40^ Although topographic heterogeneity may provide localized climatic refugia, the climatic niches of many endemic montane species are nevertheless expected to contract substantially under continued warming.^41^

Land-use change is also identified as another important determinant for terrestrial vertebrates across the globe under all four SSPs, on average resulting in 47.94% of global richness changes, which is in line with many existing studies.^42^^,^^43^^,^^44^ This suggests that land-use change also plays a significant role in shifting species ranges and changing richness diversity of terrestrial vertebrates. However, the effect of land-use change on terrestrial vertebrates often shows a superposition or mitigation effect on the effect of climate change. For instance, Jung et al.^14^ claimed that abrupt land-use change could lower local species richness and abundance by 4.2% and 2.0%, respectively, but this loss could completely recover after 10 years with a constant climate condition. Moreover, our analysis shows that land-use change, particularly in low-latitude regions, contributes more to richness changes by comparison with climate variables, such as the mean annual temperature and the mean annual precipitation. This high land-use-related contribution may largely be associated with agricultural expansion.^45^ According to the high level of projected population growth and the dietary transitions to more calories and animal-based foods, more natural land needs to be converted into agricultural land to satisfy basic food systems,^46^^,^^47^ thereby making natural habitats more fragmented and leading to species extinctions.^48^

The combined effects of climate and land-use change on terrestrial vertebrates show substantial latitudinal differences, with a large decline at low latitudes. This result is consistent with those of previous studies, which suggest a sharp biodiversity loss at low latitudes.^33^^,^^49^ However, unlike numerous studies focusing on a poleward shift of terrestrial vertebrates in the future,^10^^,^^35^^,^^36^ we warn that the richness changes at low latitudes should be paid much more attention to. The main reason is that low-latitude regions have a considerable number of species and the most abundant biological resources on the planet. For instance, the Amazon Basin is home to nearly one-quarter of terrestrial species. Besides that, the low latitudes are subject to some of the locations that are most disturbed by anthropogenic activities, including land-use change and degradation, pollution, and overexploitation. Multiple anthropogenic stressors have made tropical ecosystems more vulnerable and transformed them from species-rich systems to species-poor systems.^50^ Furthermore, our result at the country-specific level indicates that the richness loss is mainly concentrated in the countries at the middle-income level, which is highly consistent with that of the study by Waldron et al.^51^ Their study finds that biodiversity declines as the gross domestic product (GDP) grows, but the effect of GDP growth is not significant in the poorest countries and can be partly offset by improvements in the quality of national governance. Obviously, countries at different income levels have different abilities to cope with the effects of climate change, as well as varied social consciousness and paid willingness for biodiversity conservation,^52^^,^^53^ leading to different magnitudes of species-richness changes. Meanwhile, the phenomenon is also closely related to economic activities. Some other studies have shown that high-income countries can shift their pressure on species to low- and middle-income countries through importing of products and services.^54^^,^^55^ These telecoupled activities make the country-specific richness changes more complex.

Global biodiversity will be affected by both climate change and land-use change, and climate change is considered the dominant cause of species extinction. How society responds to climate change will seriously affect biodiversity changes, because effective climate change mitigation policies will significantly alleviate the direct effect of climate change on biodiversity.^43^^,^^49^ Our analysis shows that under the scenario of the highest land-use and climate pressures, namely the SSP5 scenario, the diversity of terrestrial vertebrates will decline the most by the middle of the 21st century. This demonstrates that our society must immediately implement sustainable development strategies through transforming energy production and consumption, improving renewable energy technologies, reducing greenhouse gas emissions, and slowing down the rate of climate change. What is more, reasonable land-use planning is equally important for biodiversity conservation, especially at a country-specific level. First, reducing deforestation and agricultural expansion are the most direct ways to conserve species through protecting habitats. A market-based protective payment mechanism, such as REDD+,^56^ can be employed to higher the cost of deforestation for private-sector actors. Improved agricultural production efficiency and proactive food system changes are also essential approaches to reducing biodiversity threats. Second, the establishment of protected areas and ecological networks for species at risk of extinction can help alleviate pressures associated with future land-use change. The effectiveness of global protected areas is not optimistic, as they were designed before considering the importance of management.^57^ Previous studies in Europe have demonstrated that establishing connected protected area networks can improve conservation outcomes by maintaining habitat connectivity, reducing the impacts of habitat fragmentation, and facilitating species dispersal under future land-use change.^58^ Third, establishing laws for local species is proven to be beneficial to strictly prohibit overexploitation and illegal trade of endangered species.^59^ Other channels, including newly established economic regulations like payment for ecosystem services,^60^^,^^61^ are substantial tools as financial supports for biodiversity conservation. In addition, strengthening the cooperation between science and policy at all levels is fundamental to integrate scientific, indigenous, and local knowledge to support land-use decision-making at the country-specific level.

### Limitations of the study

Several limitations should be acknowledged in this study. First, our analysis focuses exclusively on terrestrial vertebrates, which may lead to a conservative estimate of global biodiversity loss. Many invertebrate groups, particularly pollinators and other taxa with narrow niche requirements, short generation times, and strong dependence on fine-scale ecological interactions, are often sensitive to habitat fragmentation and land-use change.^62^ Therefore, the biodiversity impacts projected here may underestimate the full extent of biodiversity loss across all taxa. Future studies incorporating broader taxonomic groups and trait-based sensitivity assessments would provide a more comprehensive evaluation of biodiversity responses to global change. Second, land-use naturalness is solely represented using the biodiversity habitat index, which reflects the average capacity of landscapes to support native biodiversity. However, the lack of alternative global datasets for cross-validation may introduce uncertainty in how land-use naturalness is represented across different regions. Third, species distributions are derived from the IUCN Red List spatial range and BirdLife International spatial range databases. These species range maps may introduce uncertainty into species habitat preference and the LSI because they represent the potential geographic extent of species rather than their realized distributions. Specifically, the land-use suitability calculated from species range maps may not fully capture fine-scale variation in actual habitat use, particularly for species with restricted distributions or specialized habitat requirements. Future studies incorporating high-resolution occurrence records (i.e., citizen science observations) and species-specific habitat information could further refine habitat suitability assessments. Moreover, historical land-use information is obtained from the ESA CCI Land Cover product, while future land-use projections are based on a single global land-use modeling framework. Although these datasets provide a consistent basis for global-scale analyses, uncertainties associated with future land-use projections may propagate into biodiversity projections. Future studies should evaluate biodiversity forecasts using alternative biodiversity databases and land-use products to better quantify data-related uncertainty.

From the perspective of models, our modeling framework merely sets climate and land-use change as dominant drivers of future biodiversity change, ignoring other global change factors (i.e., nitrogen deposition, elevated atmospheric CO_2_ concentrations, invasive species, and ocean currents). In addition, owing to limited availability of globally consistent species-level data, ecological processes such as species interactions, food web alterations, and evolutionary adaptation are not incorporated into the modeling framework. Although we evaluate future species richness projections at no dispersal, limited dispersal, and unlimited dispersal assumptions, these results cannot fully represent the capacity of species to adapt through evolutionary processes or behavioral responses under global change. The omission of these drivers and biological processes may influence future biodiversity projections, particularly in regions where multiple stressors interact. Additionally, future climate projections are solely based on a CMIP6 climate model (CESM2) since it provides a comprehensive representation of coupled climate and Earth system processes. Yet reliance on a single climate model may underestimate uncertainty arising from inter-model variability in future climate conditions. Future studies integrating multi-model climate ensembles and more realistic adaptation processes could provide more robust assessments of biodiversity change under global change scenarios. Despite these limitations, our framework provides a mechanistic and spatially explicit assessment of future biodiversity change under future climate change and land-use change. Future research integrating multiple global change projections, species interactions, and adaptive responses within a unified modeling framework would improve the robustness and ecological realism of biodiversity projections.

## Resource availability

### Lead contact

Further information and requests for resources and materials should be directed to and will be fulfilled by the lead contact, Xia Li (lixia@geo.ecnu.edu.cn).

### Materials availability

This study did not generate new unique reagents.

### Data and code availability

• The terrestrial vertebrate species range maps were collected from the IUCN Red List of Threatened Species (v.2026-1, https://www.iucnredlist.org/) and Birdlife International (http://www.birdlife.org/). Historical and future climate change datasets were obtained from the WorldClim 2.1 (https://www.worldclim.org/). Historical and future land-use data datasets are available from The ESA CCI-LC (https://esa-landcover-cci.org/) and Chen et al.^63^
• All code required to reproduce the implementation of the FES framework, species distribution modeling workflow, attribution analysis, and SHAP analysis is available on Figshare: https://doi.org/10.6084/m9.figshare.33060842.
• Any additional information required to reanalyze the data reported in this paper is available from the lead contact upon request.

## Acknowledgments

This study was supported by the Key National Natural Science Foundation of China (grant no. 42130107), the National Natural Science Foundation of China (42311530335 and 42471496), the Shenzhen Science and Technology Program (JCYJ20220818100200001), and the Scientific Foundation for Youth Scholars of Shenzhen University (868-000001033431).

## Author contributions

All authors designed the study. X.L. and B.X., writing the original draft, reviewing and editing the draft, and data collection; X.L., W.T., and N.X., conceptualization, reviewing, and supervision; X.L., Y.W., and M.G., reviewing and visualization.

## Declaration of interests

The authors declare that they have no competing interests.

## Declaration of generative AI and AI-assisted technologies in the writing process

During the preparation of this manuscript, the authors used ChatGPT (GPT-5.4) to assist with language refinement. All ideas, methodologies, experiments, and conclusions were developed and verified by the authors.

## STAR★Methods

### Key resources table

**Table:** 

REAGENT or RESOURCE	SOURCE	IDENTIFIER
**Deposited data**		

Species ranges data	The IUCN Red List of Threatened Species and BirdLife International	https://www.iucnredlist.org/; http://www.birdlife.org/
Bioclimatic variables	The WorldClim 2.1	https://www.worldclim.org/
Elevation	The WorldClim 2.1	https://www.worldclim.org/
Land use data	The ESA CCI-LC and Chen et al.^63^	https://esa-landcover-cci.org/
Annual gross primary production	Wang et al.^64^ and The CMIP Phase 6	https://doi.org/10.5281/zenodo.14958479; https://wcrp-cmip.org/cmip-phases/cmip6/
Population density	WorldPop	https://www.worldpop.org/
Biodiversity habitat index	The Commonwealth Scientific and Industrial Research Organisation (CSIRO)	https://data.csiro.au/collection/csiro:54237

**Software and algorithms**		

ArcGIS 10.8	ESRI	https://www.esri.com/en-us/home
Original codes	Figshare	https://doi.org/10.6084/m9.figshare.33060842
PyCharm	Jetbrains	https://www.jetbrains.com/pycharm/

### Method details

The overall methodology of the study is illustrated in Figure S8. First, we developed a filtering-energy-stress (FES) framework to comprehensively assess land-use change according to three ecological hypotheses. This integrated framework is measured by pixel-level habitat suitability, available energy, and environmental stress. Second, we incorporated the multifaceted land-use framework and climate change into species distribution models for future global vertebrate species diversity projection. We predicted species richness of terrestrial vertebrates by using both linear and machine-learning algorithms. Models with the best performance were selected for biodiversity projection under future scenarios. Third, the combined effect of climate and land-use change on terrestrial vertebrates was assessed by projecting the species richness changes under SSPs. Finally, we evaluated the relative contribution of multifaceted land-use change for future biodiversity change at the global scale.

#### Species richness of terrestrial vertebrates

Species richness, which measures the number of different species in an ecological sample, is a biodiversity index that formed the basis for various studies.^65^ Therefore, we adopted species richness as a proxy for terrestrial vertebrate diversity in this study. Spatial distribution datasets of 7990 amphibian, 10116 reptile, and 5936 mammal species were derived from the International Union for Conservation of Nature (IUCN) Red List of Threatened Species ( v.2026–1, https://www.iucnredlist.org/). As for 11125 bird species, we adopted spatial ranges data from Birdlife International (http://www.birdlife.org/). All species range polygons were processed at Lambert Cylindrical Equal Area projection and converted to a 10 km × 10 km fishnet grids, a resolution previously validated as the finest justifiable for these data globally.^32^ Grid cells within the distribution range of each species were thus converted to presence points, while those outside their distribution ranges were converted as absence points. After excluding species with fewer than 10 raster cells, the terrestrial vertebrates comprised 5486 mammal species, 7834 reptile species, 5495 amphibian species, and 10693 bird species. Species richness was defined as the total number of species present within each grid cell. The terrestrial vertebrate diversity was calculated as species richness by summing all unique mammal, reptile, amphibian, and bird species occurring within each grid cell.

#### Land-use filtering-energy-stress framework

To comprehensively assess the multifaceted effects of land-use change on vertebrate species diversity, we developed a filtering-energy-stress (FES) framework grounded in the habitat filtering, available energy, and environmental stress hypotheses (Figure S9). For each dimension of this framework, we built a land-use suitability index (LSI), available energy index (AEI), and land-use intensity index (LII), respectively. Specifically, the LSI characterizes species-specific habitat affinity based on the habitat filtering hypothesis. This metric is measured by the degree of correspondence between the current land-use composition of a spatial grid cell and habitat preferences of a given species. In which, the land-use composition data were generated at 10 km resolution by a fine-scale European Space Agency Climate Change Initiative Land Cover (ESA CCI-LC) products. The land-use types of CCI-LC data were reclassified into seven categories, including forest, grassland, barren, cropland, urban, water, and snow/ice (Table S2). The final land-use composition products were produced by calculating the fractional coverage of each land-cover type within each 10 km grid cell. Species habitat preferences were approximated using the proportional coverage of different land-use types within each species current range. We assumed that land-cover types occupying larger proportions of a species range better reflect its broad-scale habitat associations. As IUCN range polygons describe the extent of occurrence rather than realized occupancy, this assumption provides a broad-scale approximation of habitat preference suitable for global analyses. Based on this approach, the pixel-level LSI can be calculated as follows:

(Equation 1) $${LSI}_{i}^{n}=\sum_{k=1}^{K}\left(p_{i,k}^{n}\times f_{i,k}^{n}\right)$$

Where ${LSI}_{i}^{n}$ is the land-use suitability index of species *n* for pixel *i*. For a specific species *n*, *p*_*i,k*_ is the species habitat preference percentage for land-use type *k* in pixel *i*, and *f*_*i,k*_ is the fractional coverage of land-use type *k* in pixel *i*. *K* is number of the land-use types, which is set as seven in this study. The value range of LSI is (0,1]. The LSI attains the maximum value when the pixel land-cover composition perfectly matches the species habitat preferences, and a minimum value when there is no correspondence.

To quantify ecological capacity of sustaining species richness across land-use types, we constructed an AEI according to the available energy hypothesis. In this study, we assumed that a land-use type with greater ecological integrity would provide more accessible energy resources to support species diversity. Therefore, the AEI is formulated as the product of land-use naturalness and ecosystem energy as Equation 2.

(Equation 2) $${AEI}_{i}=\log_{10}\left(E_{i}+1\right)\times \sum_{k=1}^{K}\left(N_{i,k}\times f_{i,k}\right)$$

where *AEI*_*i*_ represents the available energy of grid cell *i*. *E*_*i*_ denotes the potential energy of cell *i*, for which we adopted annual gross primary productivity (GPP) as a proxy given its strong correlation with species richness at the global scale.^21^^,^^66^ We obtained annual GPP data from Wang et al.,^64^ which provided high-accuracy estimates by using satellite remote sensing techniques. To reduce the influence of exceptionally productive regions, annual GPP was converted to logarithmic transformation. *N*_*i,k*_ denotes naturalness of land-use type *k* in grid cell *i*. The naturalness of different land-use types was derived from a biodiversity habitat index data, which quantified habitat intactness by assessing the extent to which different land-use types retain the ecological conditions necessary to support species assemblages.^67^ In this study, we assumed that the naturalness reflects the accessibility of energy resources within a specific land-use type. *f* is the fractional coverage of land-cover types within grid cells, as calculated in LII. Based on the ecological energy availability hypothesis, higher AEI values generally indicate greater potential for habitat carrying capacity to species persistence.

The LII serves to capture the magnitude of environmental stress imposed upon habitats. Given that population density is widely regarded as a primary driver of anthropogenic environmental stress,^32^ we incorporated it into the LII as a component of land-use intensity. Specifically, we assumed that areas with higher population density experience disproportionately greater environmental stress. Therefore, for a grid cell *i*, *LII*_*i*_ can be expressed as:

(Equation 3) $${LII}_{i}=\log_{10}\left({pd}_{i}+1\right)\times \sum_{k=1}^{K}\left(\left({1-N}_{i,k}\right)\times f_{i,k}\right)$$

Where *pd*_*i*_ is the population density within grid cell *i*, obtained from the WorldPop database. To diminish the influence of extremely dense population aggregation, we applied a logarithmic transformation to population density. *f*_*i,k*_ represents the fractional coverage of land-use type *k* in cell *i*, and *N*_*i,k*_ denotes the naturalness of land-use type *k* in grid cell *i*. We defined (1-*N*_*k*_) as the disturbance sensitivity of land-use type *k*. High *LII* values thus indicate severe land-use pressure, which implies an elevated risk of species persistence.

#### Species distribution models

Species distribution models (SDMs) are widely used to predict species distributions and assess their response to environmental change. The basic assumption of SDMs is that species distributions are constrained by environmental conditions, and that such relationships can be leveraged to estimate habitat suitability across space and time.^68^^,^^69^ A variety of statistical and machine-learning algorithms have been developed for SDM construction, differing in their capacity to capture complex relationships between species and habitat, and to extrapolate under new environmental conditions.^70^ For instance, generalized linear (GLM) models and generalized additive (GAM) models are well-established regression-based approaches that provide interpretable representations of species–environment relationships through response functions.^71^ In comparison, machine-learning methods such as random forests (RF) and Extreme Gradient Boosting (XGBoost) are capable of accommodating nonlinear responses and complex interactions among predictors.^72^ Given the potential for variation in predictive accuracy across algorithms, we calibrated four SDM approaches, including GLM, GAM, RF, and XGBoost, for all vertebrate species with default parameter settings. Presence data were drawn from the gridded range maps. We considered 10 grid cells as the minimum necessary to fit response curves to different environmental variables. According to the study of Newbold,^32^ pseudo-absence samples in our analysis were randomly selected from terrestrial grid cells outside the known occurrence localities of each species, with the number of pseudo-absences set to two times the number of presence records to improve model calibration. The performance of each algorithm was evaluated by the different accuracy metrics (Table S1), including the area under the curve (AUC), true skill statistic (TSS), R^2^, and Kappa. The algorithm achieving the best performance among these metrics was subsequently selected for future biodiversity projection.

For all SDMs, we incorporated historical and future bioclimatic variables from WorldClim 2.1 (Table S3), which encompass 19 metrics for seasonal and annual patterns of temperature and precipitation. To reduce multicollinearity, a principal component analysis was applied to retain the principal components that collectively explained at least 85% of the total variance. These components broadly represented climatic gradients associated with temperature and precipitation regimes (Figure S1). Future climate projections were obtained from the Coupled Model Intercomparison Project Phase 6 (CMIP6) based on the Community Earth System Model version 2 (CESM2) climate model, which has been widely applied in biodiversity projection due to its advanced representation of biogeochemical cycles and land-atmosphere interactions.^73^ Following the approach of Ruacho-González et al.,^74^ we specifically selected future climate variables covering the period 2021–2040, 2041–2060, 2061–2080, and 2081–2100, representing the time horizon of 2040, 2060, 2080, and 2100, respectively, from four SSP scenarios (SSP1, SSP2, SSP3, and SSP5). This selection encompasses the full range of plausible emissions trajectories, from stringent mitigation to high-forcing pathways, facilitating a systematic evaluation of biodiversity responses across different climate changes.^75^ We quantified the indices of habitat suitability, available energy, and land-use intensity based on a future land-use projection dataset, which was generated using a Cellular Automata (CA) model driven by the baseline ESA CCI-LC product.^63^ To maintain consistency with climate projections, we utilized land-use scenarios under SSP1-RCP1.9, SSP2-RCP2.6, SSP3-RCP7.0, and SSP5-RCP8.5. Correspondingly, future datasets for population density and gross primary productivity (GPP) were aligned with these specific SSP scenarios. In addition, mean elevation was included reflect the effect of elevation on species richness when building species distribution models. All climate, land-use and terrain variables were resampled to a 10 km spatial resolution, with Antarctic grid cells and those containing missing values excluded from subsequent analysis.

#### Contributions of land-use and climate change

To quantify the contributions of climate change and land-use change to richness changes of terrestrial vertebrates, we decomposed the total richness change in each grid cell into its two constituent drivers following an additive partitioning approach. Specifically, the spatially land-use-induced species richness (*Bio*_*land*_) was simulated by using the constant land-use dataset and future climate dataset. Similarly, the climate-driven species richness (*Bio*_*climate*_) was projected with the constant climate dataset and future land-use dataset. Here, we considered the projected species richness with future climate and land-use dataset as the actual species richness under SSPs. Accordingly, we could calculate the difference between the actual species richness and the *Bio*_*land*_, and the difference between the actual species richness and *Bio*_*climate*_ using Equations 4 and 5.

(Equation 4) $${\Delta Bio}_{\text{land}}={Bio}_{land}-{Bio}_{land\_climate}$$

(Equation 5) $${\Delta Bio}_{\text{climate}}={Bio}_{climate}-{Bio}_{land\_climate}$$

Following Wu et al.^76^ and Liu et al.,^77^ we estimated the relative contributions of climate change and land-use change to richness changes of terrestrial vertebrates as Equations 6 and 7. Here, we considered the sum of contributions of climate change and land-use change to be 100%.

(Equation 6) $${\text{Contr}}_{\text{land}}=\frac{\left|{\Delta Bio}_{land}\right|}{\left|{\Delta Bio}_{land}\left|+\right|{\Delta Bio}_{climate}\right|}\times 100\%$$

(Equation 7) $${\text{Contr}}_{\text{climate}}=\frac{\left|{\Delta Bio}_{climate}\right|}{\left|{\Delta Bio}_{land}\left|+\right|{\Delta Bio}_{climate}\right|}\times 100\%$$

The sum of these two components was constrained to equal the total projected richness change, ensuring that contributions are mutually exclusive and exhaustive. Contribution values were expressed as proportions of the absolute total change, ranging from 0 to 1, where values above 0.5 indicate driver dominance. This decomposition was applied independently across all four SSP scenarios.

To further evaluate the relative importance of specific predictors in driving species richness change, we apply SHAP analysis to the best-performing species distribution models. In general, SHAP provides a unified, model-agnostic measure of feature importance by assigning each predictor a Shapley value that reflects its average marginal contribution to the model output. For each terrestrial vertebrate species, we computed the mean absolute SHAP value of bioclimatic land-use variables and elevation to rank their relative influence on projected species richness changes. Higher mean absolute SHAP values indicate greater influence of the corresponding predictor on model outputs.

#### Uncertainty and sensitivity analysis

To evaluate the uncertainty of SDMs, we compared the performance of the four algorithms before selecting the optimal model for subsequent projections. For each species, four species distribution modeling algorithms were calibrated, including GAM, GLM, RF, and XGBoost. Models were calibrated for the baseline period using 80% of occurrence records randomly selected from the species range maps and tested with the remaining 20% of records. To improve the robustness of model selection, each algorithm was run four times, and model performance was evaluated using AUC, TSS, R^2^, and Kappa metrics (Table S1). The best-performing model was selected to project future species distribution under different SSP scenarios.

We further conducted a sensitivity analysis to test how certain methodological assumptions affect our results. Because previous studies have shown that different dispersal assumptions represent a major uncertainty in SDMs for future biodiversity projection, we took three dispersal assumptions to test the robustness of our results, following the method of Newbold et al.^7^ The presented results are based on the assumption of limited dispersal, in which a specific buffer is applied to range polygon of each vertebrate species. Given the mobility of different taxonomic groups, the dispersal buffer for mammal and bird species was set as 3 km/yr, while a dispersal buffer for amphibian and reptile species was 0.5 km/yr. These rates align with dispersal assumptions in previous studies of vertebrate responses to climate change, where they are from empirical observations of species range shifts and paleoecological evidence.^7^^,^^32^^,^^78^ Although dispersal rates vary substantially within taxonomic groups, species-specific estimates remain unavailable for most vertebrates. The limited-dispersal scenario setting for climate change enables investigation of whether and where currently suitable habitat will persist, as well as the extent to which newly suitable habitat will remain accessible to species in the future. Both aspects are of considerable relevance to *in situ* conservation planning. In addition, we presented a sensitivity analysis with two other dispersal assumptions: (a) a no-dispersal scenario, in which species were confined to their current ranges; and (b) an unlimited-dispersal scenario, in which species were assumed to migrate to suitable environmental conditions fully beyond their present distributions (Figure S10).

### Quantification and statistical analysis

Quantification and statistical analyses, including model evaluation with AUC, TSS, and Kappa are described in the method details section.

### Additional resources

This study does not include additional resources.
