# Supplementary material for: Global land-use change rivals climate change in driving projected diversity shifts of terrestrial vertebrates
Source: iScience. 2026 Aug 10;29(8):117148. doi: 10.1016/j.isci.2026.117148 (PMC13486892; doi:10.1016/j.isci.2026.117148)
Supplement: Supplementary file 1 [file mmc1.pdf]

**iScience, Volume *29***

## Supplemental information

# Global land-use change rivals climate change in driving projected diversity shifts of terrestrial vertebrates

**Xiaojuan Liu, Bingqi Xie, Wei Tu, Yang Wu, Minyi Gao, Nan Xu, and Xia Li**

# SUPPLEMENTAL INFORMATION

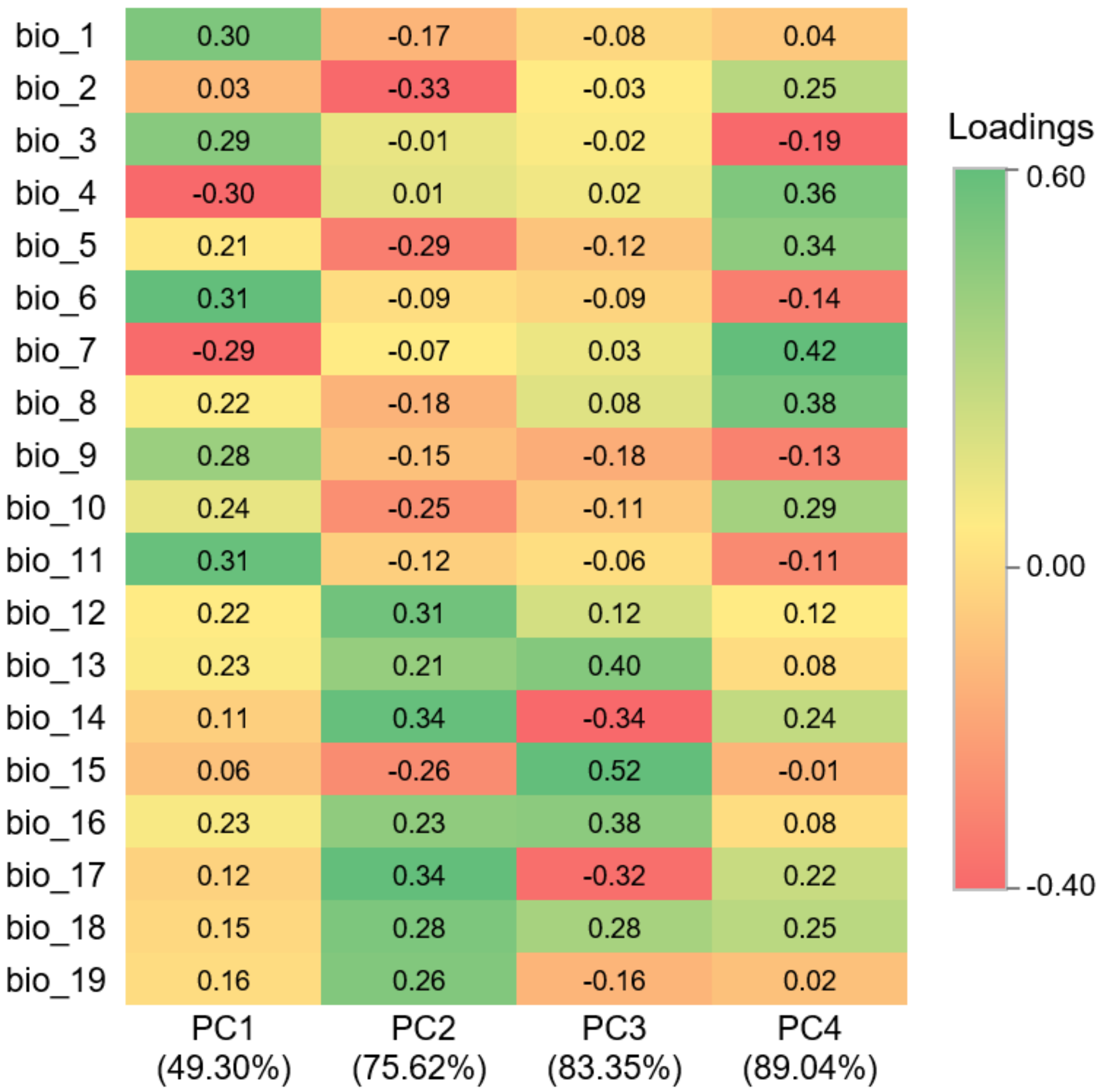


Figure S1. The loadings of principal component analysis of 19 bioclimatic variables. There are four principal components (PC) because their cumulative variance reaches beyond 85%. PC1 and PC4 largely indicate temperature gradient and extremes, respectively. PC2 and PC3 primarily represent precipitation gradient and seasonality, respectively. The definitions of bioclimatic variables are as follows: bio_1: annual mean temperature, bio_2: mean diurnal range, bio_3: isothermality, bio_4: temperature seasonality, bio_5: max temperature of warmest month, bio_6: min temperature of coldest month, bio_7: temperature annual range, bio_8: mean temperature of wettest quarter, bio_9: mean temperature of driest quarter, bio_10: mean temperature of warmest quarter, bio_11: mean temperature of coldest quarter, bio_12: annual precipitation, bio_13: precipitation of wettest month, bio_14: precipitation of driest month, bio_15: precipitation seasonality, bio_16: precipitation of wettest quarter, bio_17: precipitation of driest quarter, bio_18: precipitation of warmest quarter, bio_19: precipitation of coldest quarter.

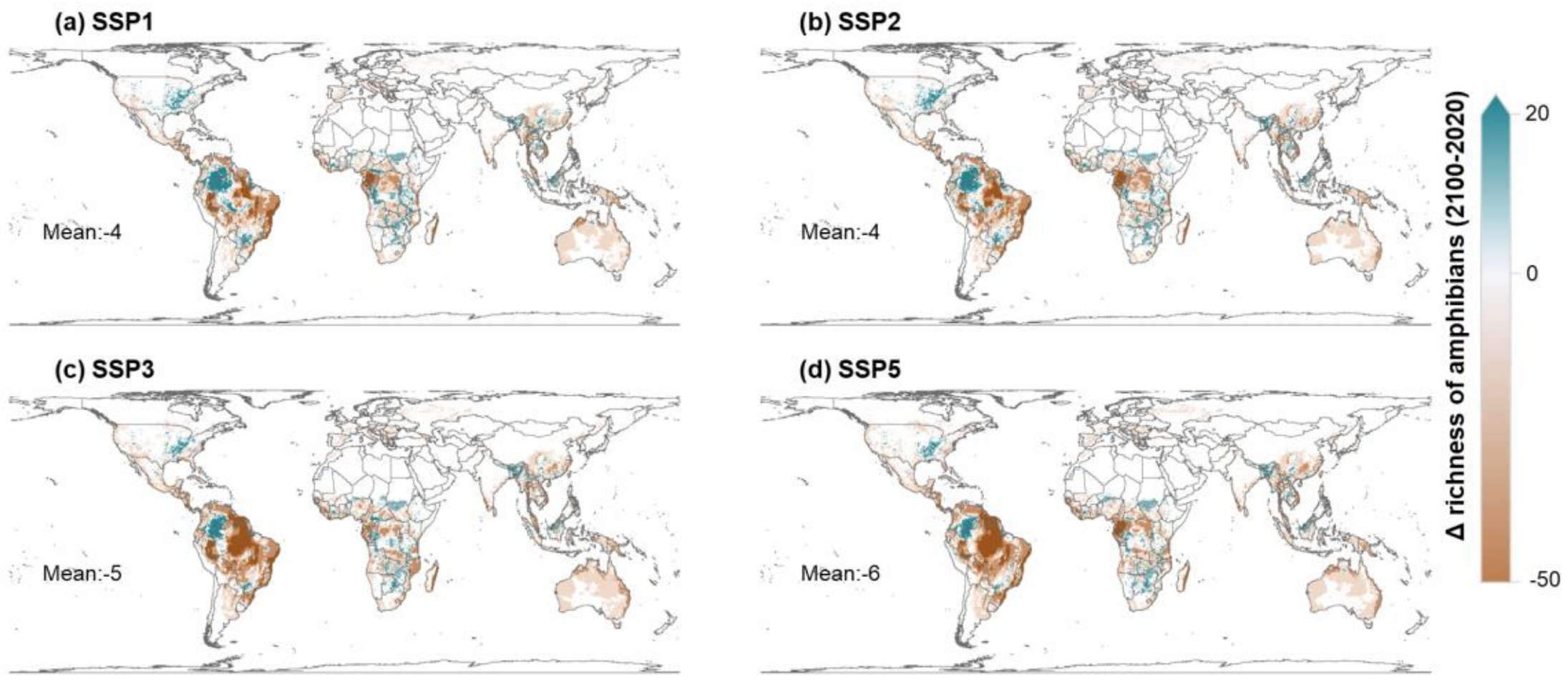


Figure S2. Richness change of amphibian species from the present to 2100 under four SSPs: (a) SSP1, (b)SSP2, (c) SSP3, and (d)SSP5. The mean value is calculated by the average species change across global grid cells.

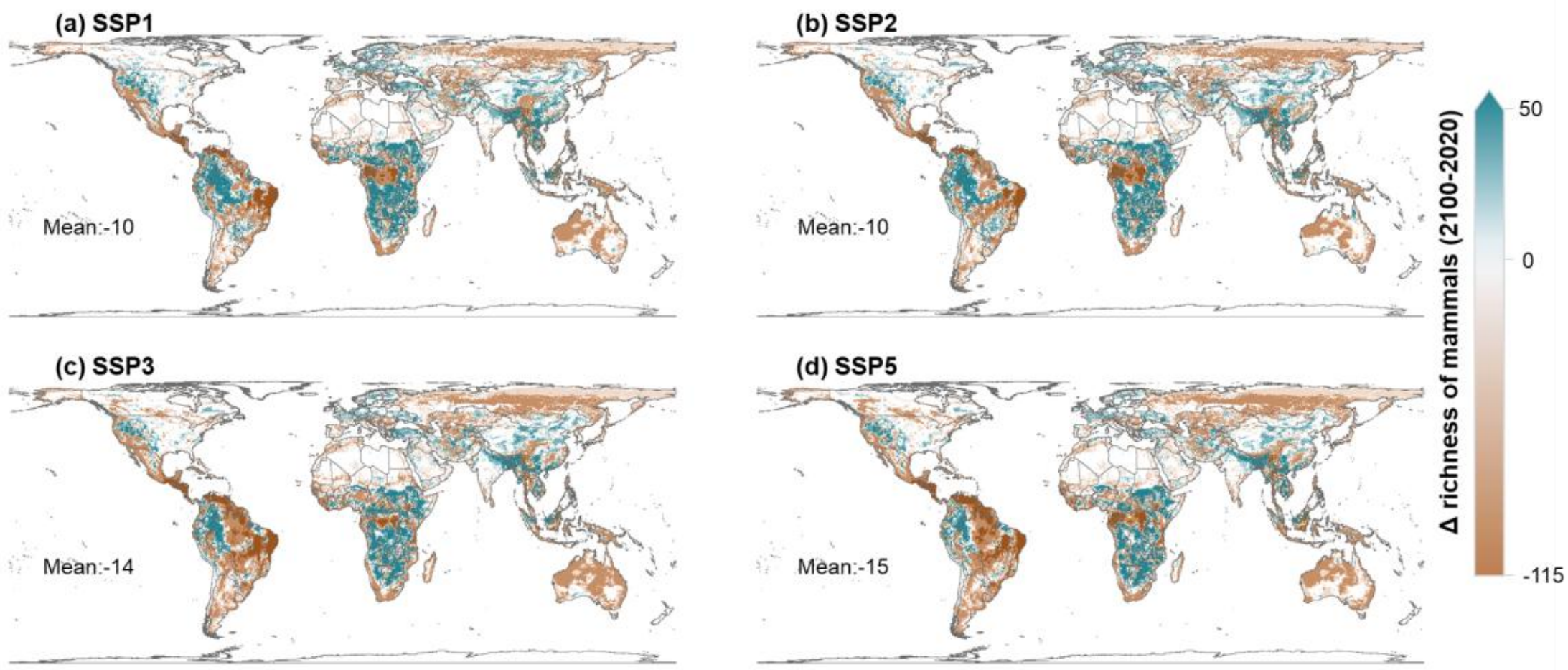


Figure S3. Richness change of mammal species from the present to 2100 under four SSPs: (a) SSP1, (b)SSP2, (c) SSP3, and (d)SSP5. The mean value is calculated by the average species change across global grid cells.

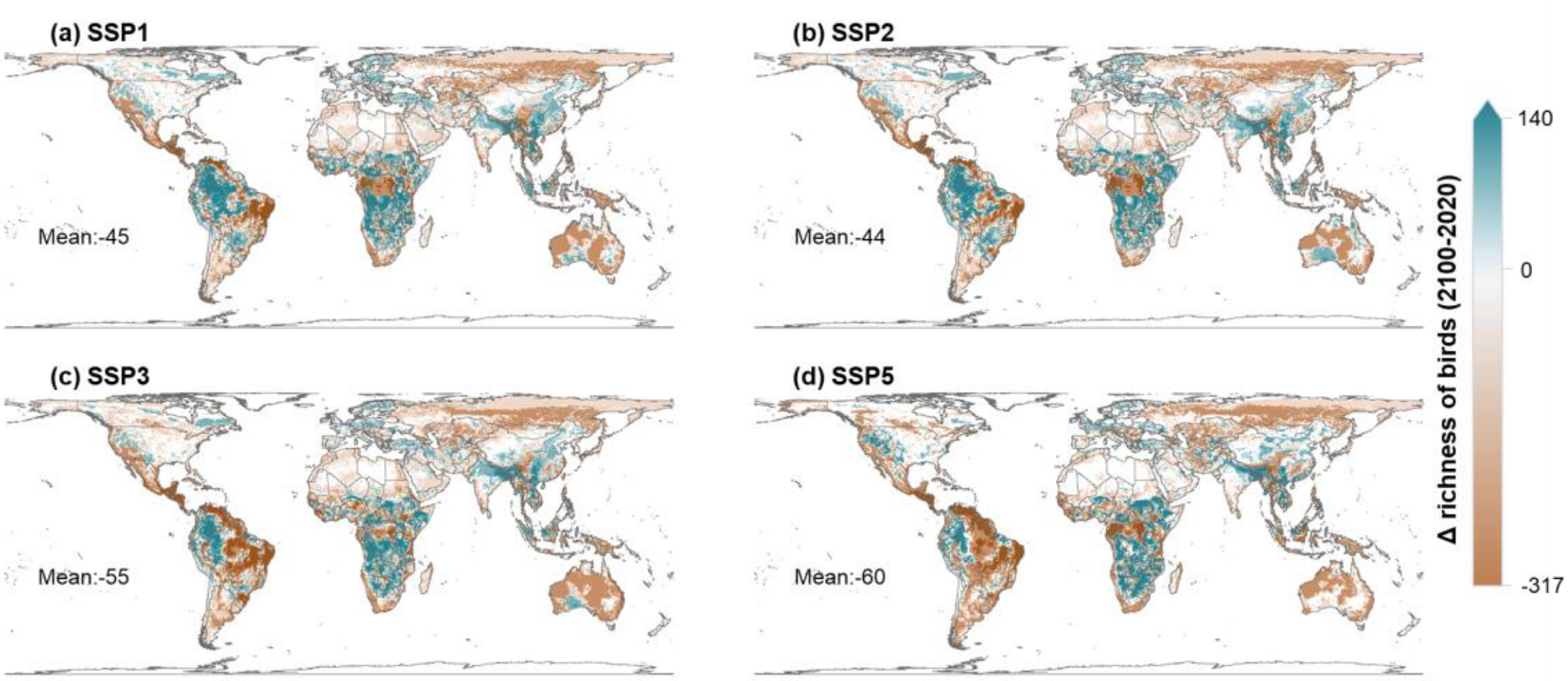


Figure S4. Richness change of bird species from the present to 2100 under four SSPs: (a) SSP1, (b)SSP2, (c) SSP3, and (d)SSP5. The mean value is calculated by the average species change across global grid cells.

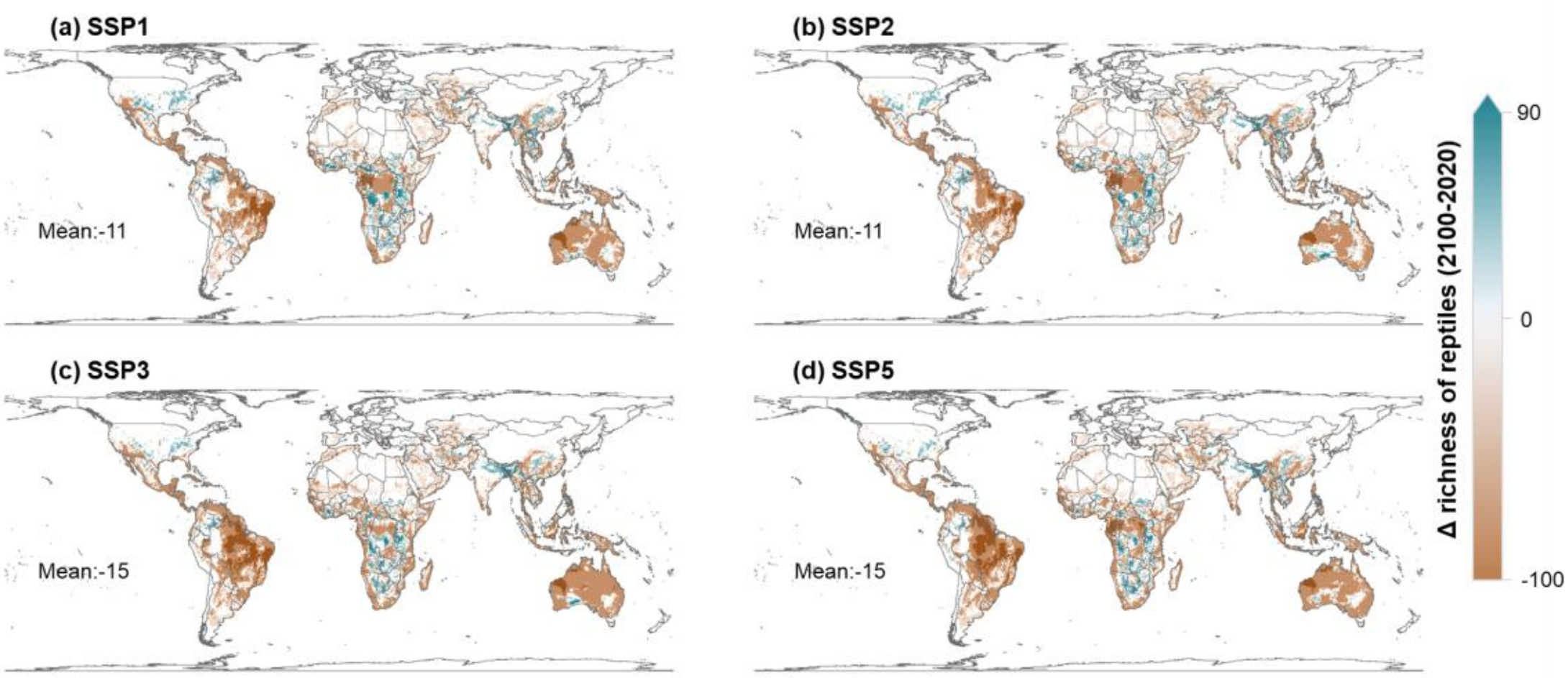


Figure S5. Richness change of reptile species from the present to 2100 under four SSPs: (a) SSP1, (b)SSP2, (c) SSP3, and (d)SSP5. The mean value is calculated by the average species change across global grid cells.

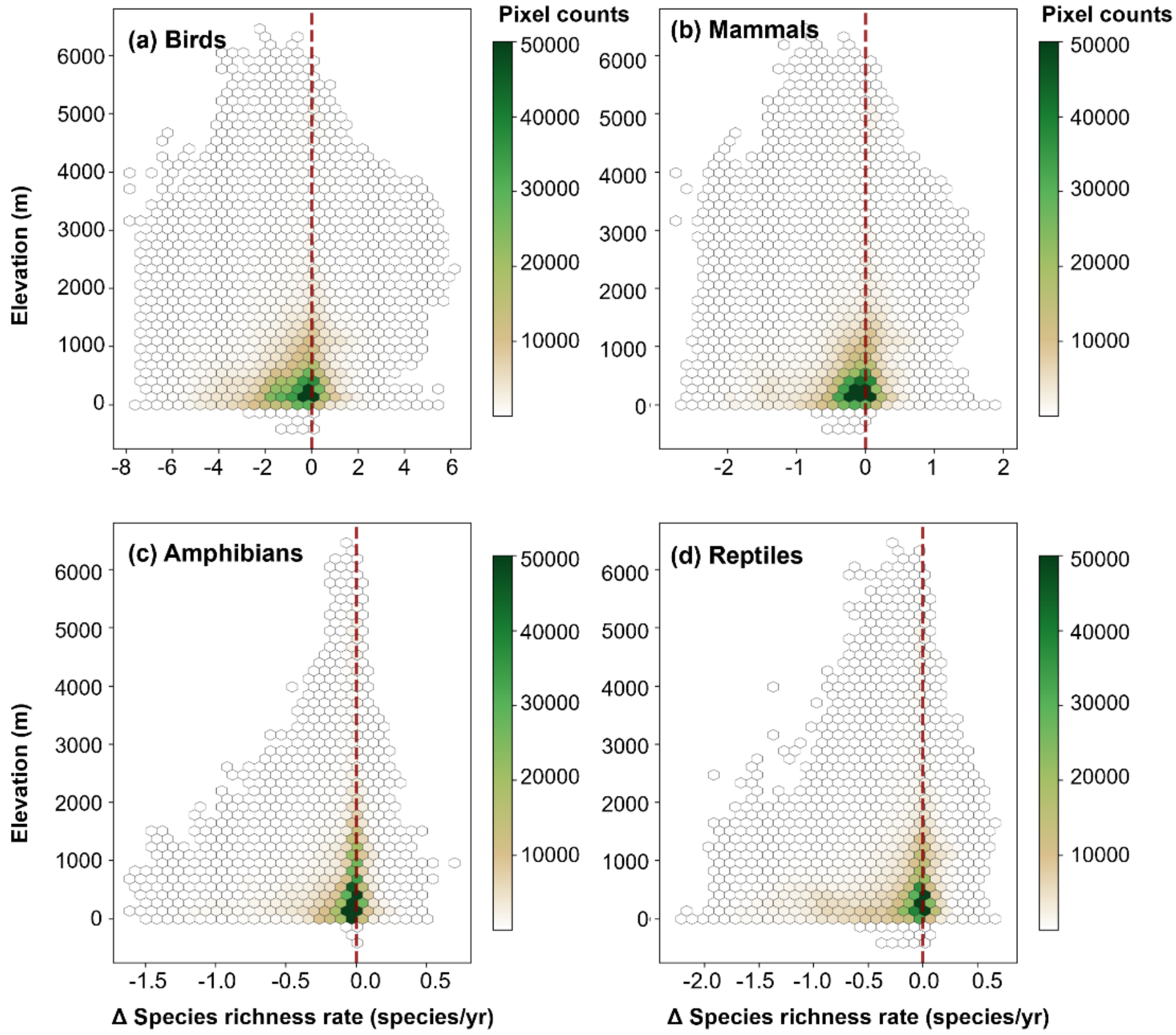


Figure S6. Annual changes of species richness in elevation between 2020 and 2100 under SSP5 for (a) birds, (b) mammals, (c) amphibians and (d) reptiles. Colour bar shows the number of grid cells that are located in the annual change of richness species and latitude.

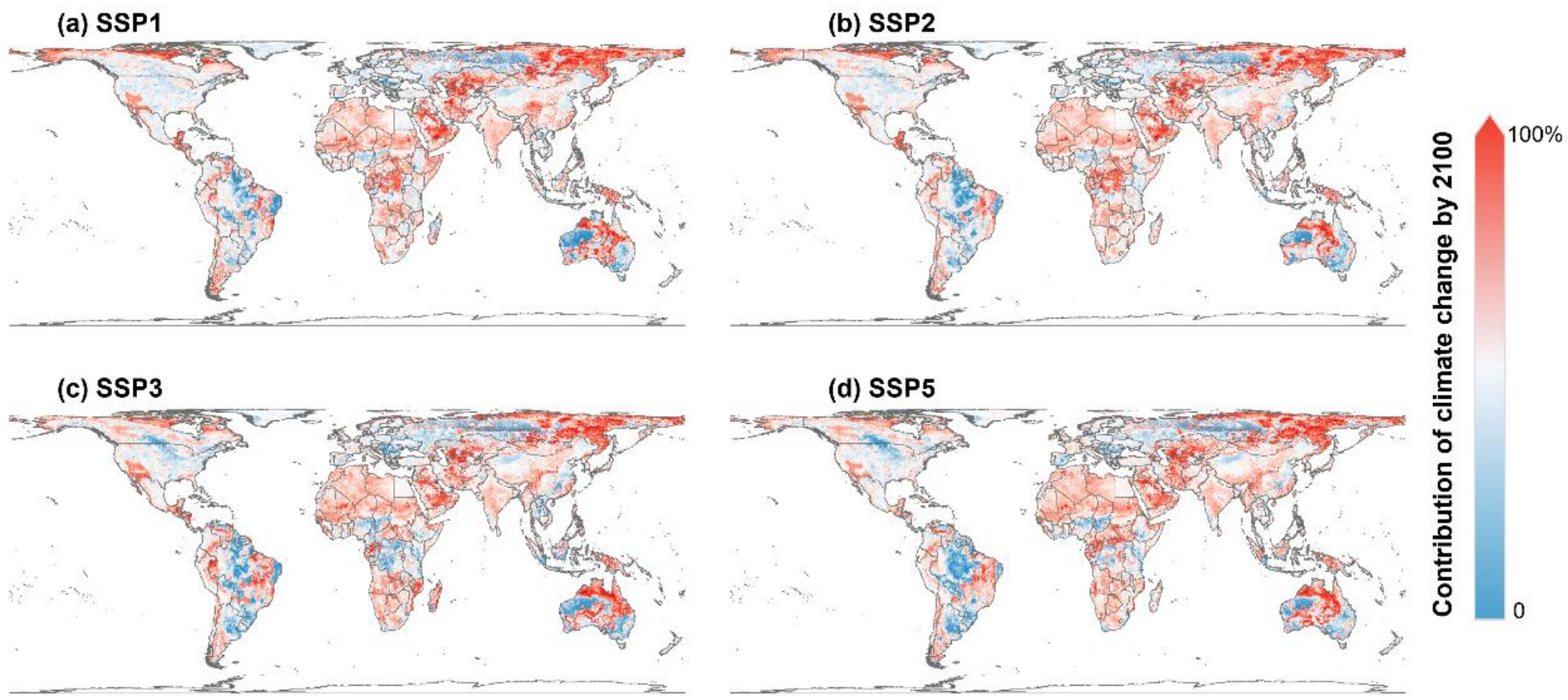


Figure S7. Contribution of future climate change on global vertebrate species changes. (a) SSP1, (b) SSP2, (c) SSP3, and (d) SSP5.

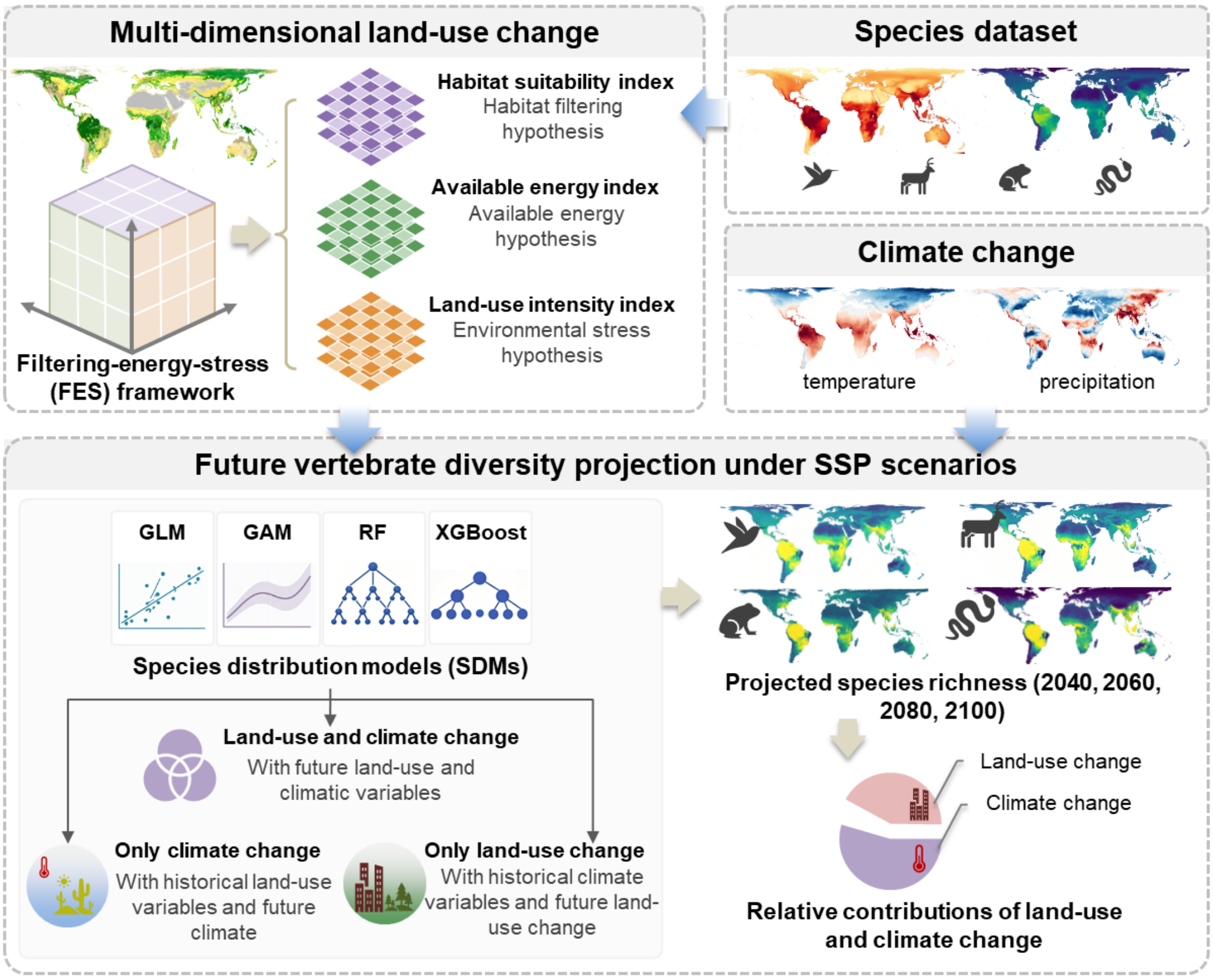


Figure S8. Flowchart of methodology for assessing the effect of land-use and climate change on terrestrial vertebrate diversity.

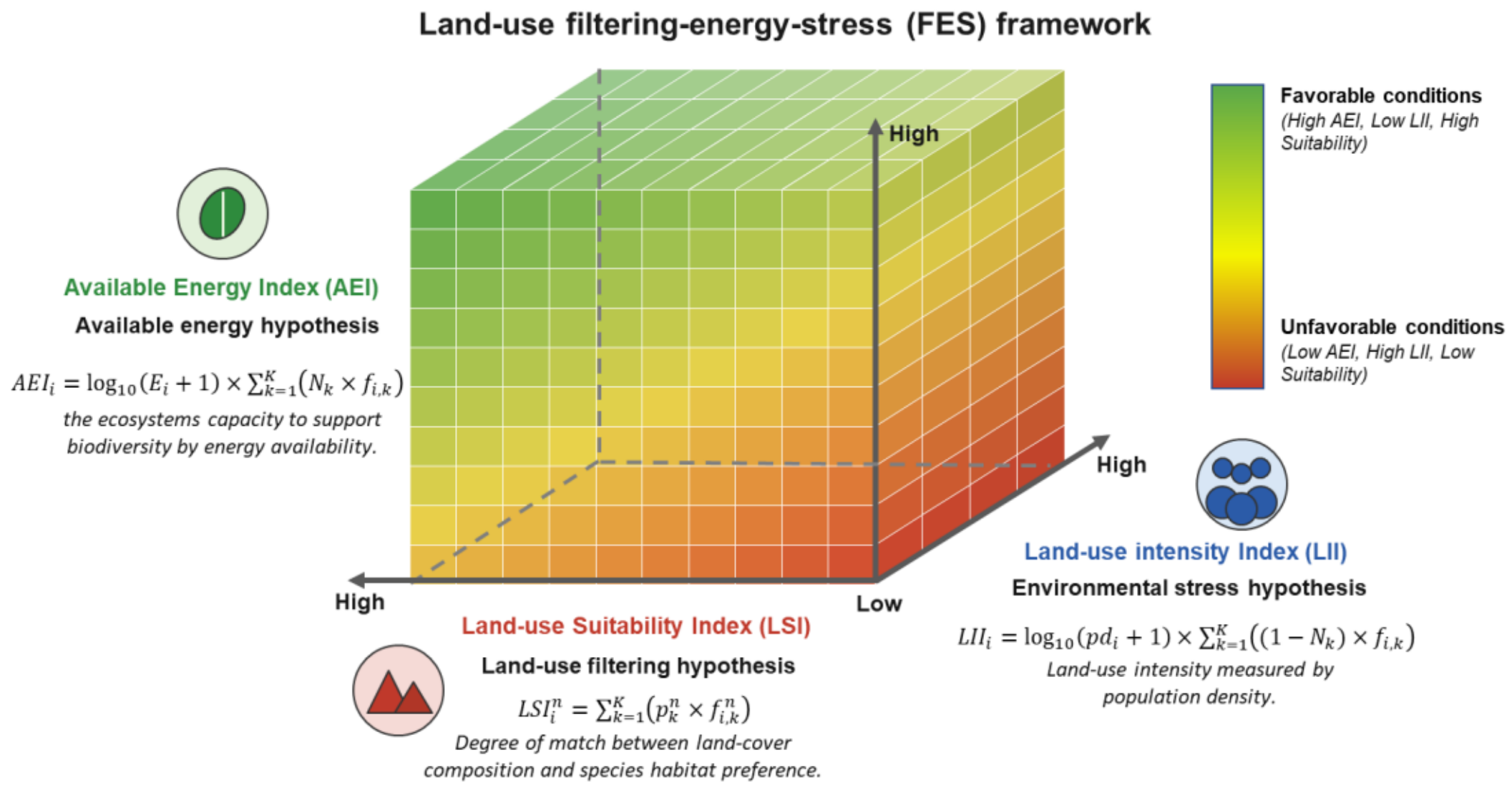


Figure S9. The integrated land-use filtering-energy-stress (FES) framework.

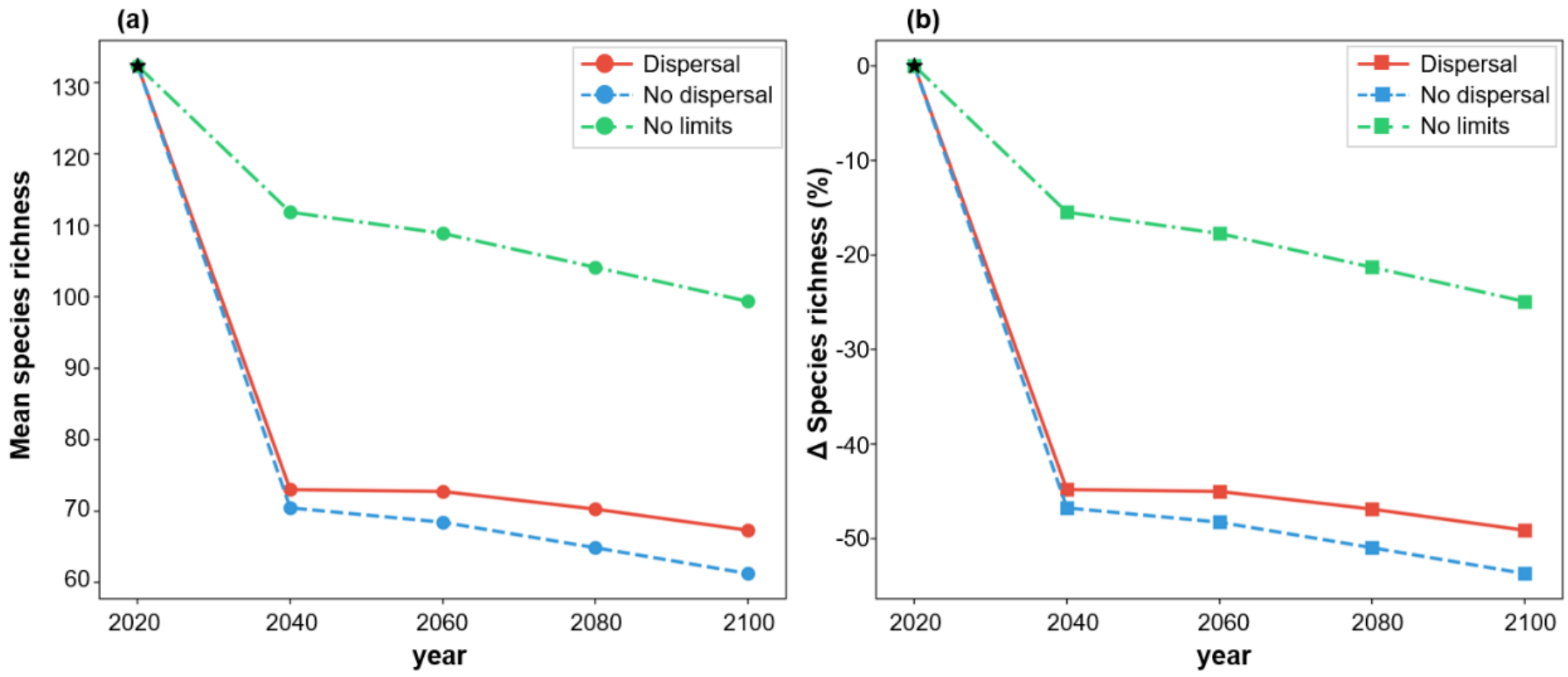


Figure S10. Species richness projection from 2020 to 2100 with limited-dispersal, no-dispersal, and unlimited-dispersal scenarios. (a) Mean species richness and (b) average richness change rate (%).

Table S1. Model performance of vertebrate species distribution projection.

| Model | Taxonomic group | Number of species | $R^2$ | AUC | TSS | Kappa |
|---|---|---|---|---|---|---|
| GAM | amphibians | 5495 | 0.8544 | 0.9919 | 0.9464 | 0.9247 |
| GAM | birds | 10693 | 0.8199 | 0.9824 | 0.8937 | 0.8970 |
| GAM | mammals | 5486 | 0.8636 | 0.9897 | 0.9261 | 0.9204 |
| GAM | reptiles | 7834 | 0.8348 | 0.9917 | 0.9344 | 0.9172 |
| GLM | amphibians | 5495 | 0.7175 | 0.9772 | 0.8353 | 0.8190 |
| GLM | birds | 10693 | 0.7048 | 0.9550 | 0.7923 | 0.8030 |
| GLM | mammals | 5486 | 0.7319 | 0.9669 | 0.8206 | 0.8234 |
| GLM | reptiles | 7834 | 0.7153 | 0.9733 | 0.8234 | 0.8141 |
| RF | amphibians | 5495 | 0.8882 | 0.9976 | 0.9085 | 0.9259 |
| RF | birds | 10693 | 0.8644 | 0.9930 | 0.8928 | 0.9083 |
| RF | mammals | 5486 | 0.8864 | 0.9958 | 0.9116 | 0.9248 |
| RF | reptiles | 7834 | 0.8853 | 0.9972 | 0.9055 | 0.9207 |
| XGBoost | amphibians | 5495 | 0.8875 | 0.9965 | 0.9127 | 0.9203 |
| XGBoost | birds | 10693 | 0.8611 | 0.9908 | 0.8940 | 0.9061 |
| XGBoost | mammals | 5486 | 0.8832 | 0.9939 | 0.9114 | 0.9194 |
| XGBoost | reptiles | 7834 | 0.8815 | 0.9950 | 0.9087 | 0.9154 |

*Note*: The performance metrics are the mean values for each taxonomic group.

Table S2. The land-use types of ESA-CCI dataset and their corresponding naturalness.

| **Land-use types (this study)** | **Land-USE types (ESA CCI-LC)** |
|---|---|
| Agriculture | Rained cropland<br>Irrigated cropland<br>Mosaic cropland (>50%)/natural vegetation (tree, shrub, herbaceous cover) (<50%)<br>Mosaic natural vegetation (tree, shrub, herbaceous cover) (>50%)/cropland (< 50%) |
| Forest | Tree cover, broadleaved, evergreen, closed to open (>15%); Tree cover, broadleaved, deciduous, closed to open (> 15%); Tree cover, needleleaved, evergreen, closed to open (> 15%); Tree cover, needleleaved, deciduous, closed to open (> 15%); Tree cover, mixed leaf type (broadleaved and needleleaved); Mosaic tree and shrub (>50%) / herbaceous cover (< 50%) |
| Grassland | Mosaic herbaceous cover (>50%) / tree and shrub (<50%); Grassland |
| Water | water |
| Wetland | Shrub or herbaceous cover, flooded, fresh-saline or brakish; Tree cover, flooded, fresh or brakish water; Tree cover, flooded, saline water |
| Urban | Urban |
| Other | Shrubland; Lichens and mosses; Sparse vegetation (tree, shrub, herbaceous cover); Bare areas |

Table S3. The dataset used for species distribution models.

| Data | Time | Resolution | Sources |
|---|---|---|---|
| Species ranges data | present | - | The IUCN Red List (https://www.iucnredlist.org/ ) and BirdLife International (http://www.birdlife.org/) |
| Bioclimatic variables | 1970-2000, 2040, 2060, 2080, 2100 | 5 arc-minutes | The WorldClim 2.1 (https://www.worldclim.org/) |
| Elevation | present | | |
| Land use data | 2020, 2040, 2060, 2080, 2100 | 1km | The ESA CCI-LC (https://esa-landcover-cci.org/) and Chen et al. [1] |
| Annual gross primary production | 2020, 2040, 2060, 2080, 2100 | 1km, 0.9375° | Wang et al.[2] and the CMIP Phase 6 (https://wcrp-cmip.org/cmip-phases/cmip6/) |
| Population density | 2020, 2040, 2060, 2080, 2100 | 1km | The WorldPop database (https://www.worldpop.org/) |
| Biodiversity habitat index | 2020 | 1km | The Commonwealth Scientific and Industrial Research Organisation ( https://data.csiro.au/collection/csiro:54237) |

*Note*: Future bioclimatic variables are obtained from WorldClim as 20-year averages (2021–2040, 2041–2060, 2061–2080, and 2081–2100), which are hereafter referred to as 2040, 2060, 2080, and 2100, respectively.

## Supplementary References


1. Chen, G., Li, X., and Liu, X. (2022). Global land projection based on plant functional types with a 1-km resolution under socio-climatic scenarios. Scientific Data *9*, 125. https://doi.org/10.1038/s41597-022-01208-6.
2. Wang, S., Huang, N., Wang, L., and Zhang, Y. (2025). Dataset of global terrestrial gross primary productivity from 2000 to 2020 (Zenodo). https://doi.org/10.5281/zenodo.14958479.
